# Modulation of CRISPR‐Cas9 Cleavage with an Oligo‐Ribonucleoprotein Design

**DOI:** 10.1002/cbic.202400821

**Published:** 2025-01-20

**Authors:** Yahui Gao, Yan Shan Ang, Lin‐Yue Lanry Yung

**Affiliations:** ^1^ Department of Chemical & Biomolecular Engineering National University of Singapore Singapore 117585 Singapore

**Keywords:** CRISPR Cas9, modulation of Cas9, DNA cleavage, DNA, genome editing

## Abstract

Clustered regularly interspaced short palindromic repeats (CRISPR) associated protein Cas9 system has been widely used for genome editing. However, the editing or cleavage specificity of CRISPR Cas9 remains a major concern due to the off‐target effects. The existing approaches to control or modulate CRISPR Cas9 cleavage include engineering Cas9 protein and development of anti‐CRISPR proteins. There are also attempts on direct modification of sgRNA, for example, structural modification via truncation or hairpin design, or chemical modification on sgRNA such as partially replacing RNA with DNA. The above‐mentioned strategies rely on extensive protein engineering and direct chemical or structural modification of sgRNA. In this study, we proposed an indirect method to modulate CRISPR Cas9 cleavage without modification on Cas9 protein or sgRNA. An oligonucleotide was used to form an RNA‐DNA hybrid structure with the sgRNA spacer, creating steric hindrance during the Cas9 mediated DNA cleavage process. We first introduced a simple and robust method to assemble the oligo‐ribonucleoprotein (oligo‐RNP). Next, the cleavage efficiency of the assembled oligo‐RNP was examined using different oligo lengths in vitro. Lastly, we showed that the oligo‐RNP directly delivered into cells could also modulate Cas9 activity inside cells using three model gene targets with reduced off‐target effects.

## Graphical abstract

Basic sgRNA structure could be modified by adding oligo DNA. The assembly of oligo‐sgRNA with Cas9 protein (oligo‐RNP) could modulate the Cas9 cleavage on on‐target and off‐target DNA. The oligo‐RNP could be further delivered into mammalian cells for improving genome editing specificity.

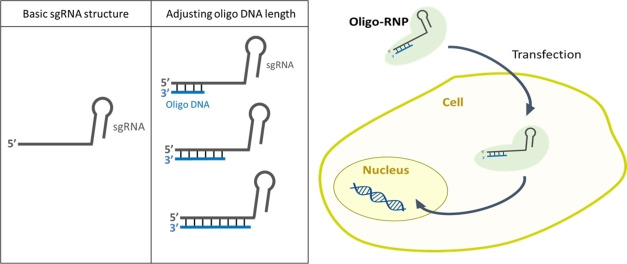

## Introduction

The clustered regularly interspaced short palindromic repeats (CRISPR) associated protein 9 (Cas9) system, or CRISPR‐Cas9, has become a favourable technique for genome editing.[^1^, ^2^, ^3^, ^4^] As an RNA‐guided genome editing, Cas9 nuclease binds directly to the single guide RNA (sgRNA) to form a ribonucleoprotein (RNP) complex. The cleaving process starts with the recognition of the short protospacer‐adjacent motif (PAM) site on the DNA target by Cas9 protein, followed by conformational change and R loop formation via hybridization of the spacer region of the sgRNA with the complementary target DNA sequence in the genome.[^5^, ^6^] CRISPR‐Cas9 system can be reprogrammed to target different sites in the genome by altering the sgRNA spacer sequence. However, the on‐target activity and off‐target effects of individual sgRNAs can vary widely as Cas9 can tolerate up to 4 PAM‐distal mismatches and high homology in seed region with one or two mismatches could also lead to off‐target events.[^6^, ^7^, ^8^, ^9^]

Multidisciplinary approaches have been proposed to modulate the CRISPR Cas9 cleavage with the aim to reduce off‐target effects. Computational approaches have been developed to optimize the 20‐nucleotide (20 nt) spacer design of the sgRNA by predicting the potential off‐target sites. These off‐target prediction algorithms are useful but are inadequate to provide a complete solution to the specificity issue.[^10^, ^11^, ^12^, ^13^] Recent approaches are focused on exploring Cas orthologues or creating engineered Cas9 variants with improved fidelity or specificity.[^14^, ^15^, ^16^, ^17^, ^18^] Anti‐CRISPR (Acr) proteins such as CRISPR‐Cas inhibitors have also been used to reduce off‐target editing by administering Acr proteins shortly after Cas9 and sgRNA delivery^[19]^ or either co‐expressing or coupling Acr with Cas9 protein.^[20]^ Several works have also been done to modify the sgRNA design structurally and chemically in order to modulate off‐target effects. sgRNA spacers could be truncated^[21]^ or engineered with secondary structures such as hairpin to impose steric hindrance during the Cas9 cleavage process in order to modulate the cleavage activity.^[22]^ Chemically, sgRNA could also be modified with 2′‐*O*‐methyl‐3′‐phosphonoacetate^[23]^ or partially replaced with DNA[^24^, ^25^] for the purpose of reducing off‐target effects. These approaches are promising, however, require extensive protein engineering work and direct structural or chemical modification on sgRNA property, and may only be specific to certain Cas orthologue or sgRNA sequences.

In this work, we propose a simple yet effective design to modulate the Cas9 cleavage activity by adding an oligo DNA with complementarity to the spacer region of the sgRNA to form an RNA‐DNA hybrid (RDH) duplex structure. The assembled DNA coupled ribonucleoprotein (RNP) (oligo‐RNP) creates a steric and energetic barrier to R‐loop propagation, which can modulate the Cas9 cleavage activity and potentially reduces the off‐target effects. This approach avoids direct modification on the structural and chemical properties of sgRNA. The RDH structure was designed intentionally to be thermodynamically less stable than the RNA‐RNA duplex but more stable than DNA‐DNA duplex. Several oligo‐RNPs with different oligo DNA lengths were designed and evaluated for their modulation effect on Cas9 cleavage efficiency. Furthermore, the oligo‐RNPs were directly delivered into cells for evaluation of the modulation effect of Cas9 cleavage activity in a cellular environment.

## Results and Discussion

### Design Considerations for Oligo‐RNP

**Figure 1:**
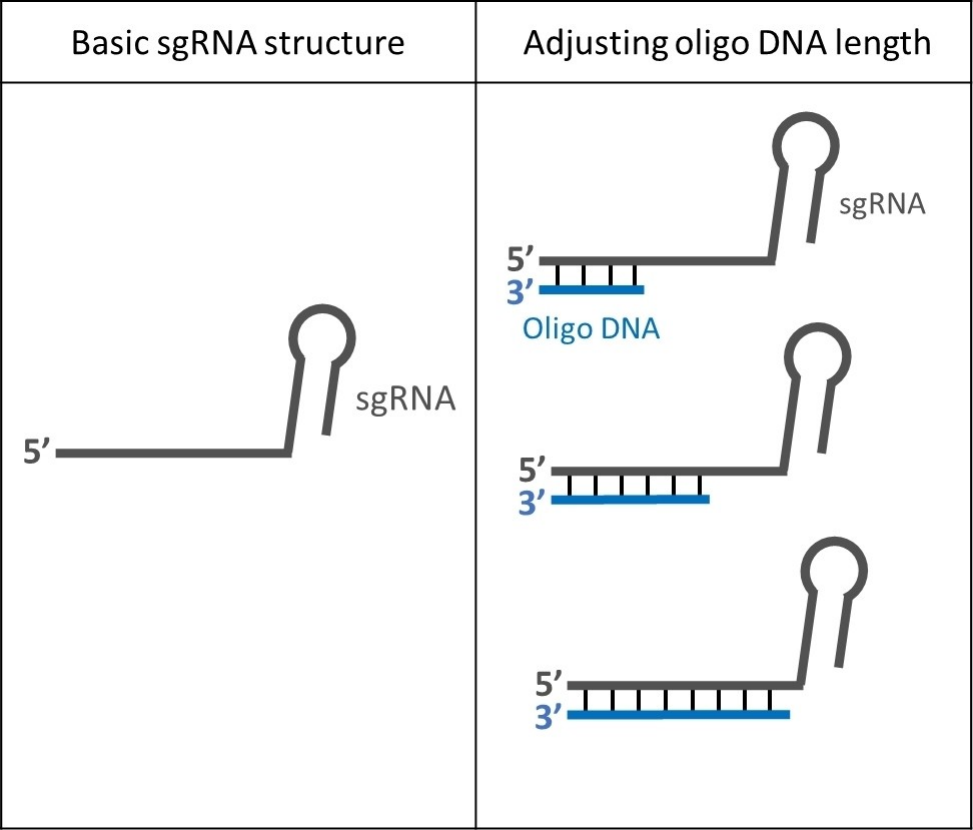
Schematic illustration of sgRNA (dark grey) and proposed oligo‐sgRNA designs with oligo DNAs of different lengths (dark blue).

Previous studies have shown that seed region on sgRNA can significantly affect the binding and cleaving activity of the CRISPR‐Cas9 system.[^1^, ^5^, ^6^, ^26^] We designed the oligo DNA to be complementary to the sgRNA spacer region starting from the PAM‐distal 5’ end (non‐seed region) and extending into part of the seed region (PAM‐proximal 10–12 nt located in the 3’ end of the sgRNA 20 nt spacer sequence) (Figure 1). As the length of the oligo DNA increases, the oligo DNA is expected to form RDH structure covering both non‐seed region and part of the seed region of the sgRNA spacer.

As a proof of concept, sgRNA sequence for gene target Empty Spiracles Homeobox 1 (EXM1) was used to test our oligo designs in vitro. The 20 nt EXM1 sgRNA sequence and its on‐target and off‐target sequences have been reported and well characterized previously.[^21^, ^22^] The melting temperature (T_m_) and guanine‐cytosine content (GC content) of the RDH of different lengths were examined. Ideally, the T_m_ should be high enough so that the RDH structure is stable at room temperature as well as at 37 °C in a cellular environment. If the oligo length is too short, the RDH would not be formed stably. GC content is closely related to T_m_; if the oligo DNA length is the same, higher GC content increases the corresponding T_m_. In this study, T_m_ was estimated using an online tool, OligoAnalyzer (Integrated DNA Technologies (IDT)) as a guide for choosing the oligo length. As shown in Table 1, the estimated T_m_ for a RDH structure of 6 nt is between 6.2 °C and 10.9 °C, indicating that the RDH duplex will not be formed. 9 nt RDH has an estimated T_m_ between 37 °C and 40 °C, suggesting possible incomplete formation of the RDH. Therefore, 9 nt was selected to be the minimal nucleotide length in this study. In the subsequent experiments, we investigated the following four nucleotide lengths, 9 nt, 12 nt, 15 nt and 18 nt. A full list of oligo DNA sequences and the spacer sequences of sgRNAs used in the study can be found in the Table S1.

**Table 1:** Melting temperatures (T_m_) and GC content of EXM1 oligo DNA designs. T_m_ for RNA‐RNA and DNA‐DNA duplexes were estimated by OligoAnalyzer (IDT). The NaCl concentration set for both RNA‐RNA and DNA‐DNA was 300 mM. Concentration of oligos were kept at 1 μM.

EXM1 (Sequence complementary to sgRNA spacer)		T_m_ of RNA‐RNA (°C)	T_m_ of DNA‐DNA (°C)	GC content
6nt	5′ GGACTC 3′	6.2	10.9	66.7%
9nt	5′ CTCGGACTC 3′	37.8	40.3	66.7%
12nt	5′ CTGCTCGGACTC 3′	55.0	55.8	66.7%
15nt	5′ CTTCTGCTCGGACTC 3′	62.7	61.5	60.0%
18nt	5′ CTTCTTCTGCTCGGACTC 3′	68.1	65.3	55.6%

### Formation of the Oligo‐RNP Complex

**Figure 2:**
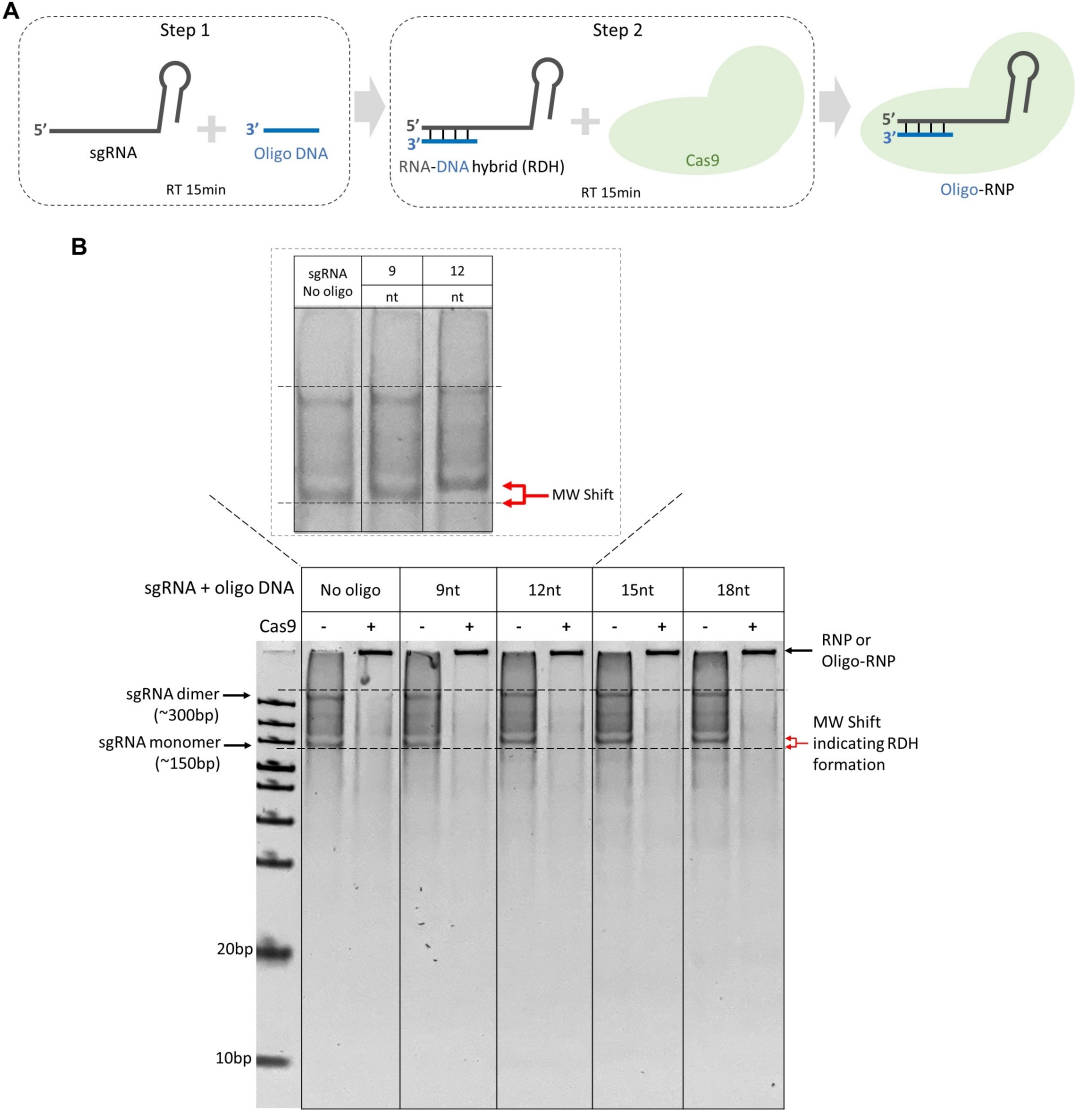
Formation of oligo‐RNP. (A) Schematic illustration of the method for assembly of oligo‐RNP complex. RDH structure is formed via hybridization of sgRNA and oligo DNA in step 1, followed by step 2 in which Cas9 protein is added to interact with the formed RDH for the formation of final oligo‐RNP. RT stands for room temperature. (B) Gel electrophoresis image of sgRNA, sgRNA with RDH or oligo‐RNP complex formed. 9nt, 12nt, 15nt and 18nt represent different oligo designs used in formation of RDH with sgRNA. Dotted box shows the gel result for sgRNA without oligo DNA and sgRNA with RDH using oligo 9nt and 12nt in which the molecules were migrated for a longer time with better separation. “No oligo” means no oligo was added. “–”and “+” represent conditions without Cas9 or with Cas9 added. In the conditions where Cas9 was not added (“–”), sgRNA dimer refers to the dimer structure formed by 2 sgRNA monomers via hybridization between their secondary structures (non‐spacer regions). The shift in mobility of RDH formed via hybridization of oligo DNA and sgRNA is indicated by arrows in red. When Cas9 was added (“+”), the bands on top were indicated as RNP or oligo‐RNP formed by RDH and Cas9.

We assembled the RDH with the widely used *Streptococcus pyogenes* Cas9 (SpCas9). As shown in Figure 2A, the oligo DNA and sgRNA were annealed to form the RDH first before Cas9 was added for interaction with the RDH to complete the assembly of oligo‐RNP complex. Figure 2B shows the result of the formation of RDH using sgRNA and different oligo DNA designs, 9 nt, 12 nt, 15 nt and 18 nt. The successful formation of RDH was indicated by a shift in mobility of both sgRNA monomer and dimer on the 10 % polyacrylaymide gel (black dotted lines and red arrow) since oligo DNA‐sgRNA with RDH structure had a slightly larger molecular weight compared to the sgRNA alone. The sgRNA dimer structure consistently observed in our gel result was supported by NUPACK simulation (Figure S1),^[27]^ in which the ratio of sgRNA monomer to dimer was estimated to be 4 : 1. In the condition when Cas9 protein was not added, based on the shift of the sgRNA monomer bands, RDH formation was observed in all the oligo DNA designs with complementary sequences at 12nt, 15nt and 18nt. The 9 nt oligo DNA had the smallest molecular weight, thus the shift in the corresponding RDH was less obvious than the other oligo DNA designs. Nonetheless, the small shift caused by RDH formed by oligo design 9 nt could be observed when the molecules in PAGE gels were further seperated (shown in the dotted box in Figure 2B and Figure S2). When the Cas9 protein was added, the DNA bands indicating sgRNA or oligo‐sgRNA (RDH) disappeared along with the appearance of a strong band observed in the loading well, suggesting the successful formation of larger complex between sgRNA or oligo‐sgRNA with Cas9 (~162 kDa). Although it was expected that Cas9 RNP or oligo‐RNP would accumulate in the loading wells of high‐percentage PAGE, the other strong indication suggesting the oligo‐RNP formation was that no oligo DNA bands were observed between 10‐basepair (bp) and 20 bp positions in the gel, indicating that the interaction between RDH and Cas9 protein did not displace the oligo DNA from the sgRNA (Figure S3).

In addition, a few variant oligo designs with overhangs were also tested. 3 nt non‐complementary overhangs were added on either 5’ or 3’ end of the oligo DNA (Figure S4A). The sequence of the overhang was designed to be 3 adenines (3a) or 3 cytosines (3c), representing purine and pyrimidine. The oligo DNA design with 12 nt complementary to sgRNA spacer and a 3a overhang located at the 5’ oligo DNA and proximal to non‐spacer region of sgRNA was abbreviated as “12_3a_in”. The 3a overhang located at the 3’ of the oligo DNA and distal to non‐spacer region of sgRNA as abbreviated as “12_3a_out”. The naming for other oligo DNA designs, 9 nt and 15 nt, adopted the similar pattern. The total length of the oligo DNA designs was maintained within 18 nt. Similarly, the MW shift shown on the gel picture suggests RDH with oligo 12 nt, 15 nt variants with overhangs could form the oligo‐RNPs with Cas9 protein regardless of the nucleotide composition or position of the overhang. In Figure S4B, the two oligo DNA bands for 12_3a_in and 12_3c_in were shown as reference bands on the lower right corner of the gel for direct comparison. Similar to the results shown in Figure 2, if the oligo DNA was displaced from sgRNA after Cas9 was added, a DNA band would appear at the similar location between 10 bp and 20 bp as the reference bands and with similar band intensity. In Figure S4B and S4 C, no oligo DNA bands were observed in the lanes with Cas9 added, confirming the successful formations of oligo‐RNPs using oligo 12 nt, 15 nt and their overhang variants. Overall, both oligo DNA with and without overhangs could form the oligo‐RNP with Cas9. Crystal structure studies have revealed that spCas9 in complex with sgRNA and target DNA was stabilized mainly by hydrogen bonding and hydrophobic interactions,[^6^, ^28^] which also would be the contributing forces for the formation of oligo‐RNP complex between RDH and Cas9. Previous studies also found that sgRNAs with secondary structure at the spacer region could still form RNP complex with SpCas9,[^22^, ^29^] which supports the possibility that the RDH formed on sgRNA spacer would not disrupt overall RNP formation.

### In vitro Cleaving Assays on the Oligo‐RNP Assemblies

**Figure 3:**
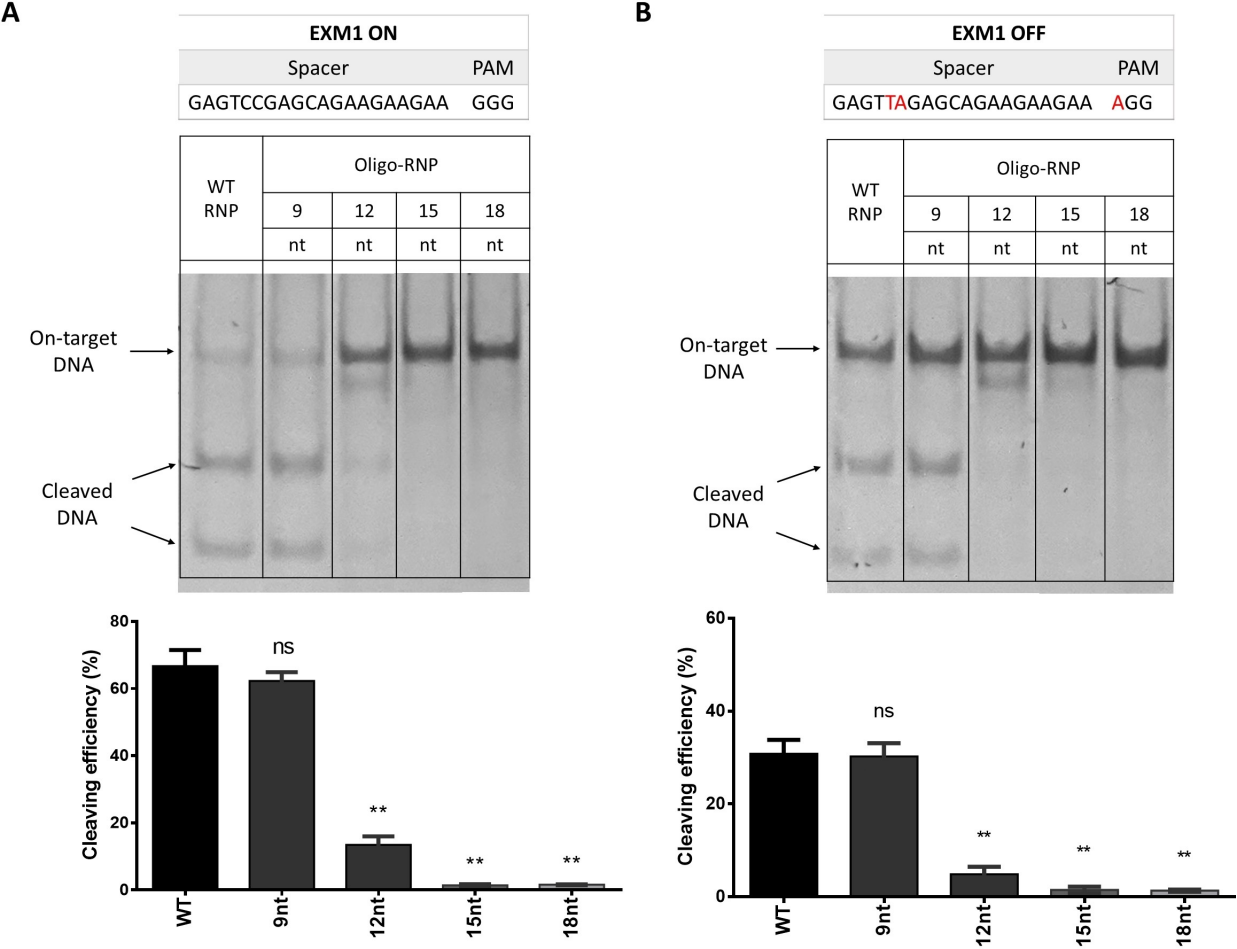
In vitro EXM1 on‐target and off‐target cleaving assay results. (A) Representative gel electrophoresis image and the corresponding quantification of the in vitro on‐target cleaving efficiencies of oligo‐RNPs. The sequence of the on‐target DNA was shown in the table on top. (B) Representative gel electrophoresis image and the corresponding quantification of the in vitro off‐target cleaving efficiency. The sequence of the off‐target DNA was shown in the table on top. Cleavage efficiency was assessed by gel electrophoresis and measured using densitometry (%).^[2]^ All data shown are mean±standard deviation (SD). Dots are individual data points from independent experiments (n=3). Oligo‐RNPs with oligo DNA designs 12 nt to 18 nt significantly reduced both on‐target and off‐target cleaving efficiencies compared to WT RNP (p<0.01). Oligo‐RNP with oligo DNA 9nt did not reduce on‐target and off‐target cleaving efficiency (ns). Statistical analysis was done using two‐tailed unpaired Student's t‐test of comparison between WT and oligo‐RNP of each oligo design. **denotes p<0.01. WT RNP is unmodified RNP without oligo DNA. All samples and controls were run on the same gel. Rearrangements of lanes in (A) and (B) can be found in the full gel images in Figure S5.

After establishing the method to assemble the oligo‐RNP using Cas9, sgRNA and the oligo DNA designs with complementary sequence 9 nt, 12 nt, 15 nt 18 nt as well as the designs variants with overhangs, the next step was to assess if the oligo‐RNP could modulate Cas9 activity in vitro using in vitro cleaving assays. Functional oligo‐RNPs were expected to modulate Cas9 activity by inhibiting Cas9 cleaving in vitro (Figure 3).

The result of in vitro cleaving assay using EXM1 on‐target DNA is shown in Figure 3A. No significant decrease in on‐target cleaving efficiency using oligo‐RNP with 9 nt oligo DNA was found compared to WT RNP. A similar result was observed for the in vitro off‐target cleaving (Figure 3B), where oligo‐RNP with 9 nt oligo DNA also did not reduce off‐target cleaving compared to WT RNP. Oligo‐RNPs with oligo DNA designs of at least 12nt complementary sequence (12nt, 15nt, 18nt) exhibited much lower cleaving efficiencies against both on‐target and off‐target EXM1 DNA compared to WT RNP. This observation suggested that the RDH design with at least 12 nt complementary sequence to sgRNA spacer (12 nt, 15 nt, 18 nt) could modulate Cas9 activity by inhibiting Cas9 cleaving in vitro. Similarly, the variant designs, 12 nt and 15 nt with overhangs (3a_in, 3a_out, 3c_in and 3c_out), also showed reduced on‐target and off‐target cleaving compared to WT RNP (Figure S5). In the subsequent investigation in cells, oligo‐RNP designs with 12 to 18 nt oligo DNA were used.

### In vivo Genome Editing of Target EXM1 using Oligo‐RNPs

**Figure 4:**
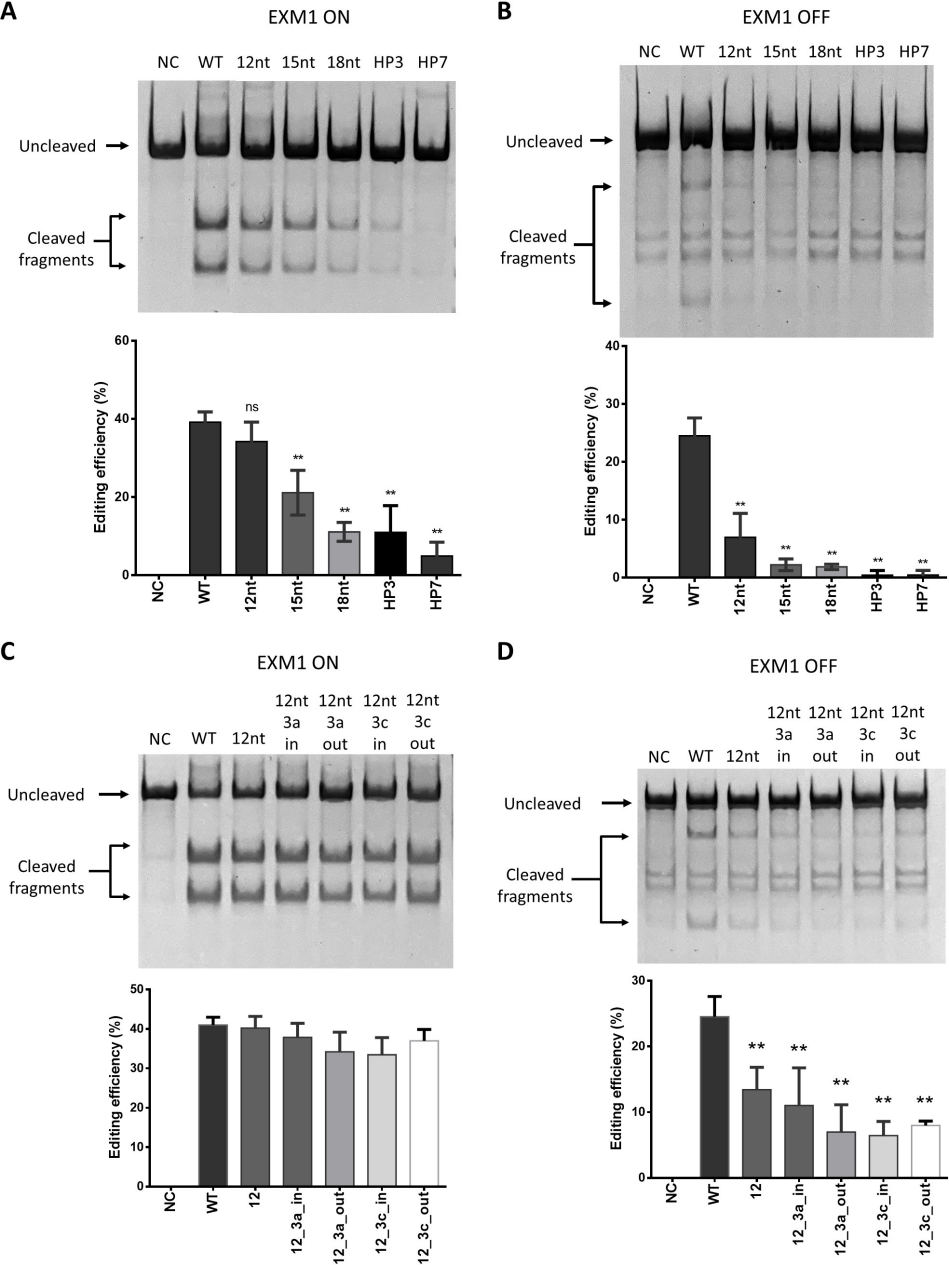
On‐target and off‐target editing efficiencies of oligo‐RNPs for EXM1 in HEK293 cells. Representative gel electrophoresis image and the corresponding quantification of (A) on‐target (ON) and (B) off‐target (OFF) editing efficiencies for EXM1 using WT RNP, oligo‐RNPs with different oligo designs (12nt, 15nt and 18nt) and RNPs with hairpin sgRNA (HP3 and HP7). * denotes p<0.05 and ** denotes p<0.01 for two‐tailed unpaired Student's t‐test of comparison between WT and oligo‐RNP of each oligo design or RNPs with sgRNA. NC is negative control. Representative gel electrophoresis image and the corresponding quantification of the (C) on‐target (ON) and (D) off‐target (OFF) editing efficiencies for EXM1 using WT RNP and oligo‐RNPs with different 12 nt oligo designs with or without overhangs. ** denotes p<0.01 for one‐way ANOVA comparison between WT and oligo‐RNP of each oligo design (12nt, 12nt_3a_in, 12nt_3a_out, 12nt_3c_in or 12nt_3c_out). NC is negative control. All quantification data shown are mean±standard deviation (SD) (n≥3). All samples and controls were run on the same gel. Rearrangements of lanes in (A) and (B) can be found in the full gel images in Figure S8. There were no rearrangements of lanes in (C) and (D).

With the in vitro study demonstrating the functionality of the oligo‐RNP complex, we then evaluated the effect of oligo‐RNP on in vivo genome editing efficiencies in mammalian cell lines for EXM1. First, the performance of oligo‐RNPs with different lengths of the oligo DNA (12nt, 15nt and 18nt). Figure 4A and 4B show the results for in vivo on‐target and off‐target editing of EXM1, respectively. As expected, oligo‐RNPs with 12 nt designs had the least impact on‐target editing efficiency as compared to WT RNP but on‐target editing in 15nt and 18 nt oligo‐RNP transfected cells were significantly reduced (Figure 4A). All oligo‐RNPs designs (12nt, 15nt and 18nt) significantly reduced off‐target editing efficiencies (Figure 4B). Two hairpin sgRNA designs (hairpin 3 and 7) for EXM1 selected from a previous study were used to compare with our oligo‐RNP designs.^[22]^ The synthetic form of the two hairpin sgRNAs for RNP‐based editing was used. Both hairpin designs have RNA‐RNA duplex formed at the 5’ end of the sgRNA spacer region, hairpin 3 (HP3) has 3 base pairs in the stem and hairpin 7 (HP7) has 5 base pairs in the stem while both have the same loop of 4 nucleotides (Figure S6). A much lower on‐target editing efficiency by HP7‐RNP was observed while HP3‐RNP complex had a similar on‐target editing as oligo‐RNP with oligo 18nt (Figure 4A). For off‐target editing, both hairpin designs (HP3 and HP7) showed decreased off‐target cleaving for EXM1 (Figure 4B). This comparison between the RDH design and hairpin sgRNA confirmed that while both could significantly reduce off‐target editing, RDH oligo‐RNP (especially oligo DNA designs 12nt and 15nt) could modulate Cas9 cleavage in cells with less inhibition on on‐target editing in cells than the previously reported two hairpin sgRNA designs.

Furthermore, the oligo designs with and without overhangs were compared using the oligo with same 12 nt complementary sequence to the sgRNA spacer region for EXM1. From Figure 4C, there was no significant difference in the on‐target editing efficiencies between the WT RNP and different variants of oligo‐RNP with length of 12 nt. For off‐target editing efficiencies (Figure 4D), all oligo‐RNPs using oligo 12 nt (with and without overhangs) had significantly lower off‐target editing efficiencies compared to WT RNP. No difference between the on‐target editing efficiencies of oligo‐RNP using oligo 12 nt without overhang and those with different overhang designs (3a_in, 3a_out, 3c_in and 3c_out) was observed. Furthermore, the oligo‐RNPs prepared using oligo design 15nt with and without overhangs (3a_in, 3a_out, 3c_in and 3c_out) were tested for their editing efficiencies in cells compared to WT RNP (Figure S7). There was no significant difference among the on‐target editing efficiencies of oligo‐RNP using oligo 15nt with and without different overhang designs (3a_in, 3a_out, 3c_in and 3c_out) but the 15 nt oligo‐RNPs generally had a lower on‐target editing efficiency compared to WT designs (Figure S7A). For off‐target editing efficiency, all oligo‐RNPs with and without overhangs significantly reduced off‐target cleaving (Figure S7B).

### In vivo Genome Editing of Target VEGFA and HBB using Oligo‐RNPs

**Figure 5:**
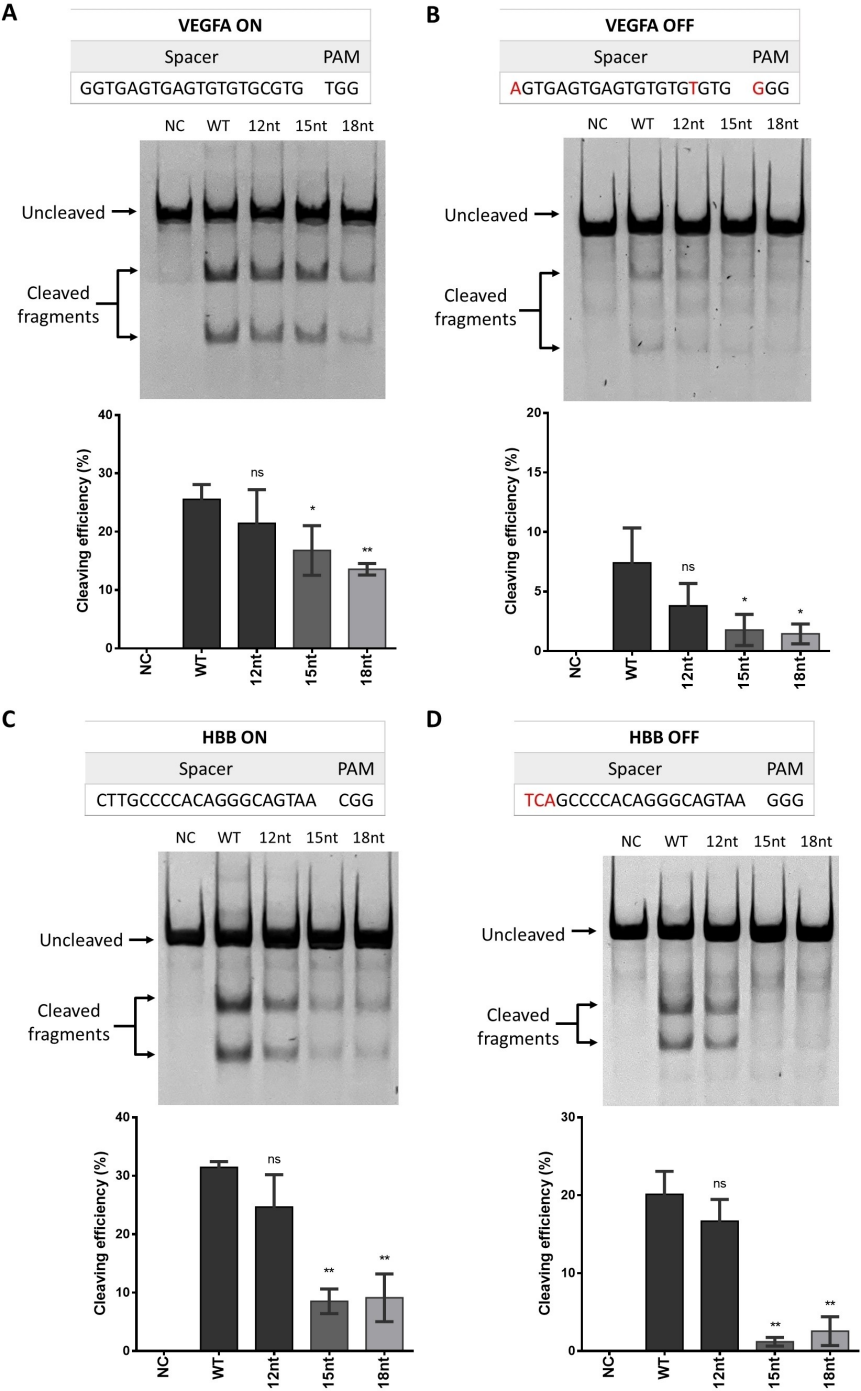
On‐target (ON) and off‐target (OFF) editing efficiencies for VEGFA and HBB in HEK293 cells. Representative gel electrophoresis image and the corresponding quantification of the (A) on‐target (ON) and (B) off‐target (OFF) editing efficiencies for VEGFA using WT RNP and oligo‐RNPs with different oligo designs (12 nt, 15 nt and 18 nt). Representative gel electrophoresis image and the corresponding quantification of (A) on‐target (ON) and (B) off‐target (OFF) editing efficiencies for HBB using WT RNP and oligo‐RNPs with different oligo designs (12nt, 15nt and 18nt). All data shown are mean±standard deviation (SD) (n≥3). * denotes p<0.05 and ** denotes p<0.01 for two‐tailed unpaired Student's t‐test of comparison between WT and oligo‐RNP of each oligo design. NC is negative control. The PAM strand of on‐target DNA has the same sequence as sgRNA spacer region. Nucleotides in red represent the mismatches on off‐target sites. All samples and controls were run on the same gel. Rearrangements of lanes in (A) and (B) can be found in the full gel images in Figure S9. Rearrangements of lanes in (C) and (D) can be found in the full gel images in Figure S10.

We have demonstrated that oligo‐RNPs with oligo length from 12nt to 18nt for target EXM1 reduced off‐target editing while having less inhibition on the on‐target editing compared to hairpin‐sgRNA designs and the inclusion of overhangs had little impact on the performance of oligo‐RNPs. To further evaluate the general applicability of our designs, another two targets, vascular endothelial growth factor A (VEGFA) and haemoglobin subunit beta (HBB), were used to examine the editing efficiency of oligo‐RNPs (Figure 5).

For VEGFA, a significant reduction in on‐target editing in oligo‐RNP transfected cells for lengths 15 nt and 18 nt was observed but not in cells transfected with oligo‐RNP with 12nt (Figure 5A). However, for off‐target editing, only oligo‐RNPs with 15 nt and 18 nt significantly reduced the off‐target effects while 12nt only slightly reduced the off‐target effects (Figure 5B). Unlike EXM1 and VEGFA, HBB sgRNA has a high GC content in the spacer region which caused the GC content of oligo 15 nt and 18 nt for HBB to be higher than that of oligo 15nt and 18nt for EXM1 and VEGFA (Table S2). Thus, oligo‐RNPs with oligo DNA 15 nt and 18 nt were expected to cause much greater reduction in on‐target editing of HBB in cells. Figure 5C shows the on‐target editing result for HBB, as expected significant reduction in on‐target editing was observed in cells transfected with oligo‐RNPs with oligo 15 nt and 18 nt, respectively. For off‐target effects, again oligo‐RNPs with oligo 15nt and 18nt significantly reduced the off‐target editing efficiencies (Figure 5D).

**Figure 6:**
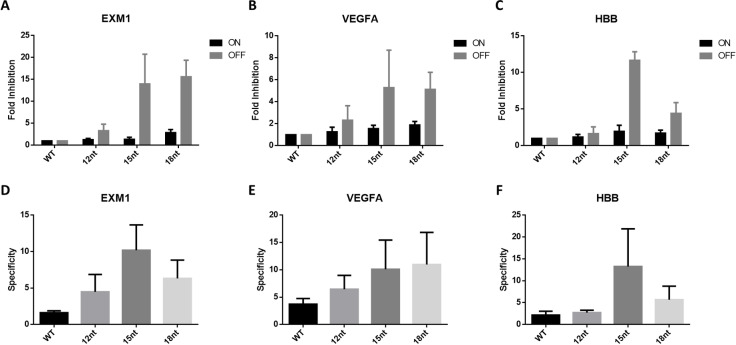
Summary of fold inhibitions for On‐target and off‐target editing of EXM1, VEGFA and HBB. (A) On‐target (ON) and off‐target (OFF) fold inhibition for EXM1. (B) On‐target (ON) and off‐target (OFF) fold inhibition for VEGFA. (C) On‐target (ON) and off‐target (OFF) fold inhibition for HBB. Fold inhibition of editing was calculated by normalizing on‐ and off‐target editing efficiencies of oligo‐RNPs with each oligo design to those of the corresponding WT RNP. (D) Specificity of editing for EXM1. (E) Specificity of editing for VEGFA. (F) Specificity of editing for HBB. Specificity was calculated by normalizing on‐target editing efficiencies with off‐target editing efficiencies for RNPs (WT or oligo‐RNPs). All data shown are mean±standard deviation (SD) (n≥3). (Note: the y‐axis (fold inhibition and specificity) scales differently among the plots).

Figure 6A to 6 C summarise the fold inhibitions of editing of oligo‐RNP mediated on‐target and off‐target editing for the three gene targets used in this study. On average, less than 2‐fold on‐target inhibition by oligo designs 12nt for EXM1, VEGFA and HBB was observed. Oligo 15nt and 18nt, however, resulted in 2 to 4‐fold inhibition for on‐target editing. For off‐target editing, much higher inhibition by oligo 15nt and 18nt for all the 3 targets (>4‐fold) was found. In terms of specificity (defined as on‐target editing normalized by off‐target editing, Figure 6D to 6F), an overall gain in specificity in all 3 targets when the length of the oligo DNA increased from 12nt to 15nt. A decrease in specificity for oligo design 18nt was observed in EXM1 and HBB results, which is possibly due to the lower on‐target editing by oligo 18 nt design. Our results inevitably follow the commonly observed phenomenon about CRISPR editing – trade‐off between the gain in specificity by reducing off‐target editing and decrease in on‐target editing. In our case, we observed higher degree of inhibition in on‐target editing along with an increase in length of the oligo DNA. With oligo DNA at 15 nt long as a reference, oligo DNA less than 15nt, such as length starting from 12 nt, generally compromises less in the on‐target editing efficiency but may have a limited reduction in the off‐target editing efficiency. To achieve the improved specificity, oligo length ranging from 12 nt to 15 nt is recommended. This observation in our study could provide certain flexibility in designing the oligo DNA based on different sgRNA spacer sequences as it grants users more control over the on‐target editing reduction.

The inhibition could be attributed to two reasons based on previous studies. The first reason is that the RDH hinders the binding of Cas9 to target DNA. As the length increases (e. g., 12 nt to 18 nt), the oligo DNA is expected to form RDH structure covering both non‐seed region and part of the seed region of the sgRNA spacer. The seed region has been reported to be critical for the interaction between Cas9‐RNP and DNA target.^[26]^ Therefore, increasing RDH can possibly decrease the binding of Cas9 to target DNA and lead to lower editing efficiency. The other reason might be the possible steric hindrance to R‐loop propagation created by RDH, which is supported by our observation that when the RDH structure was at the same length (assuming the length effect on Cas9 binding to target DNA is small), those with a lower free energy would impose a higher hindrance to R‐loop formation and RNA strand invasion during Cas9 cleaving.^[6]^ Previous kinetic modelling studies have shown that a mismatch alone could introduce an energy barrier and increase the probability of R‐loop collapse.[^30^, ^31^] In our case, the RNA‐DNA hybrid structure could have structurally hindered the R‐loop progression which served as an additional energy barrier preventing the formation of a fully open R‐loop. Previous structural studies have found that the mismatch tolerance in Cas9 off‐target activity is mainly contributed by formation of non‐canonical base pairs such as wobble pairs and Hoogsteen base pair.[^9^, ^32^] And PAM‐distal mismatches were found capable of causing the duplex unpairing, which could structurally perturb the conformational activation in the Cas9.^[9]^ In our study, the addition of the Oligo DNA at the PAM‐distal region of the sgRNA could have prevented the formation of the non‐canonical base pairs and maintained the unpairing condition especially for off‐targets.

A previous study that used the hairpin structure at the 5’ end of the sgRNA spacer showed significantly improved genome editing specificity in plasmid‐based CRISPR system.[^22^, ^33^] However, this approach involves screening of multiple hairpin‐sgRNA designs for one single target and may not be suitable for RNP‐based genome editing since Cas9 complexed with engineered hairpin sgRNA shows low *in vitro* cleaving activity,^[22]^ which has also been confirmed in our study. This can be attributed to the high thermodynamic stability of RNA‐RNA interaction in the hairpin structure.[^34^, ^35^, ^36^, ^37^] Other recent approaches to reduce the off‐target effects include an aptamer inhibitor containing a PAM sequence and 5 nt complementary to the PAM proximal seed sequence on sgRNA spacer to compete with target DNA for binding with Cas9,^[38]^ which essentially follows the concept of lowering editing efficiency by reducing binding of Cas9 RNP to the target DNA. It's worth noting that in contrast to this mentioned aptamer‐based inhibitor, the oligo DNA in our study was not used as a competitive inhibitor for Cas9 protein in the reaction, instead it was preassembled in a RNP complex. Another study introduced a chimeric RNA‐DNA guide by substituting the RNA partially with DNA, which destabilized the interaction between spacer RNA and target DNA by partially converting RNA‐DNA hybridization to DNA‐DNA hybridization in order to reduce off‐target editing.^[39]^

We expect that the stability of oligo‐RNP would be similar to that of normal RNP due to their similar biophysical properties. Studies have shown that in vivo genome editing via RNP‐based delivery could begin within 4 hours of delivery and complete in 1 day in cells,^[40]^ leading to “fast editing” and avoiding the possible genome integration commonly found in plasmid‐based delivery methods. Some clinical studies and trials have been using the RNP‐based delivery in their cell therapies.[^41^, ^42^] A potential limitation is that the delivery method for the oligo‐RNP in this study relies on commercial lipid nanoparticles, which have variable delivery efficiencies depending on the target cell types. To further improve the delivery efficiency, other delivery systems such as cationic polymers,[^43^, ^44^] viral‐like particles (VLPs), or electroporation,[^45^, ^46^] can be considered.

## Conclusions

In this study, we proposed a simple method, assembling an oligo DNA with RNP to modulate CRISPR Cas9 activity without direct modification on Cas9 protein or sgRNA. The functionality of the assembled oligo‐RNP was tested in the in vitro cleaving assay. The length of the oligos studied was adjusted while the length of the sgRNA spacer was kept constant to avoid complexities introduced by modifying the sgRNA. The successful inhibition of in vitro cleaving by our oligo‐RNPs at oligo length ranging from 12nt to 18nt proved the oligo‐RNP structure was stable and functional. The results on genome editing in cells further confirmed the modulation of editing efficiency by oligo‐RNPs, where fold inhibitions of editing were confirmed using oligo‐RNPs from 12 to 18 nt with improved editing specificity. Furthermore, we expect to see a combination of different approaches such as implementing our design in other Cas9 variants with high fidelity or specificity to further optimize the efficiency and specificity of Cas9‐based genome editing.

## Experimental Section

### Oligonucleotides and Cas9 Protein

All DNA oligonucleotides and sgRNAs used in this study were purchased from Integrated DNA (IDT) in desalted form. The sequences are provided in Table S1. Alt‐R® CRISPR‐Cas9 sgRNA from IDT were used in this study.

### Preparation of Oligo‐RNP and Electrophoretic Mobility Shift Assay (EMSA)

For Empty Spiracles Homeobox 1 (EXM1) and vascular endothelial growth factor A (VEGFA), sgRNA was mixed with oligo DNA in equimolar manner at 500 nM in 1× Phosphate Buffered Saline (PBS) (pH 7.4) at room temperature for 15 min for the formation of oligo‐sgRNA. For HBB, sgRNA was mixed with oligo DNA in equimolar manner at 2 μM in 1× PBS with 100 mM NaCl added. The mixture was then heat‐denatured at 95 °C for 5 min and re‐annealed by gradually decreasing the temperature from 95 °C to 25 °C at ramp rate of 0.1 °C/second. For RNP or oligo‐RNP, the Cas9 was incubated with sgRNA or oligo‐sgRNA in equimolar manner at 500 nM in 1× PBS (pH 7.4) at room temperature for 15 min. The samples were run on a 12 % (w*/*v) non‐denaturing polyacrylamide gel (29 : 1 acrylamide*/*bisacrylamide, BioRad) in 0.5× Tris‐borate EDTA (TBE) electrophoresis buffer. After electrophoresis, the gels were visualized by SYBR Gold gel staining (Thermo Fisher Scientific).

### In vitro Cleavage Assay

RNP or oligo RNP were prepared in the same way as stated above. The cleavage reaction was performed by incubating RNP or oligo‐RNP (150 nM) with on‐target or off‐target DNA (30 nM) in 1× NEBuffer™ 3.1(New England Biolabs) for 1 hour at 37 °C. 0.5 μL of Proteinase K (P8107, New England Biolabs) was then added into each reaction to digest the Cas9. The reaction was incubated at 37 °C for 10 min. The samples were then run on a 10 % TBE (w*/*v) non‐denaturing polyacrylamide gel in 0.5× TBE electrophoresis buffer. The quantification of the gel image was done using ImageJ.^[47]^ The in vitro cleaving efficiency was determined by the following formula:

$$Cleaving\;efficiency=\;\frac{density\;of\;cleaved\;fragments}{\begin{array}{c} density\;of\;cleaved\;fragments+\\ density\;of\;uncleaved\;target\;DNA\;fragment \end{array}}$$

### Cell Transfection

HEK293 cells purchased from ATCC were grown in Eagle's Minimum Essential Medium (EMEM), ATCC) with 10 % Fetal bovine serum (FBS) at 37 °C under 5 % CO_2_. Oligo‐RNPs were prepared in equimolar manner in 1× PBS and diluted in 12.5 μL of Opti‐MEM medium (Thermo Fisher Scientific). 1.2 μL of Lipofectamine™ RNAiMAX Transfection Reagent (Thermo Fisher Scientific) was prepared in 12.5 μL of Opti‐MEM medium separately. The RNPs or oligo‐RNPs were added to the diluted Lipofectamine™ RNAiMAX region. The 25 μL mixture was then incubated at room temperature for 15 min to form the RNP or Oligo‐RNP and lipofectamine complexes. The complexes were then transferred into a 96‐well plate. The reverse transfection was done by adding about resuspended 2–30000 cells into each well. The final concentration for the RNP or oligo‐RNP transfected per well is 10 nM. After the transfected cells were incubated for 48 h, the cells were collected for genomic DNA extraction. The extraction was done according to the manufacturer's instructions (Monarch^®^ Genomic DNA Purification Kit, New England Biolabs).

### T7E1 Cleavage Assay

Extracted DNA was diluted with H_2_O for PCR amplification. 1 ng/μL of genomic DNA was used for each PCR reaction. PCR was performed by Q5^®^ High‐Fidelity DNA Polymerase (New England Biolabs). The primer sequences and Tm information used in this study are shown in Appendix A. 5 μL of PCR products were then diluted in 1X NEBbuffer 2 (New England Biolabs) (final volume 10 μL) to be heat‐denatured, re‐annealed for heteroduplex formation using a thermal cycler according to the manufacturer's instructions. 5 units of T7 Endonuclease I (T7E1) (New England Biolabs, M0302) was added into the 10 μL heteroduplex sample for incubation for 20 min at 37 °C. The reaction was stopped by adding 0.75 μl of 0.25 M Ethylenediaminetetraacetic acid (EDTA). The samples were then run on a 5 % TBE (w*/*v) non‐denaturing polyacrylamide gel in 0.5× TBE electrophoresis buffer. The quantification of the gel image was done using ImageJ.^[47]^ Cas9‐mediated editing efficiency (percentage of indels) is calculated based on the following formula^[2]^ :

$$Fraction\;cleaved=\;\frac{density\;of\;cleaved\;fragments}{\begin{array}{c} density\;of\;cleaved\;fragments+\\ density\;of\;uncleaved\;target\;DNA\;fragment \end{array}}$$

$$\mathrm{Editing}\;\mathrm{efficiency}\;\left(\%\mathrm{indel}\right)=\;100\;\times \left[1-\sqrt{\left(1-fraction\;cleaved\right)}\right]$$

Fold inhibition of editing was calculated by normalizing on‐ and off‐target editing efficiencies of oligo‐RNPs with each oligo design to those of the corresponding WT RNP. Specificity was calculated by normalizing on‐target editing efficiencies with off‐target editing efficiencies for RNPs (WT or oligo‐RNPs).

## Conflict of Interests

The authors declare no conflict of interest.

1

## Supporting information

As a service to our authors and readers, this journal provides supporting information supplied by the authors. Such materials are peer reviewed and may be re‐organized for online delivery, but are not copy‐edited or typeset. Technical support issues arising from supporting information (other than missing files) should be addressed to the authors.

Supporting Information

## Data Availability

The data that support the findings of this study are available from the corresponding author upon reasonable request.

## References

[1] M. Jinek , K. Chylinski , I. Fonfara , M. Hauer , J. A. Doudna , E. Charpentier , Science. 2012, 337, 816. 22745249 10.1126/science.1225829 PMC6286148

[2] L. Cong , F. A. Ran , D. Cox , S. Lin , R. Barretto , N. Habib , P. D. Hsu , X. Wu , W. Jiang , L. A. Marraffini , F. Zhang , Science. 2013, 339, 819–823. 23287718 10.1126/science.1231143 PMC3795411

[3] W. Jiang , D. Bikard , D. Cox , F. Zhang , L. A. Marraffini , Nat. Biotechnol. 2013, 31, 233–239. 23360965 10.1038/nbt.2508 PMC3748948

[4] P. Mali , L. Yang , K. M. Esvelt , J. Aach , M. Guell , J. E. DiCarlo , J. E. Norville , G. M. Church , Science. 2013, 339, 823–826. 23287722 10.1126/science.1232033 PMC3712628

[5] M. Jinek , F. Jiang , D. W. Taylor , S. H. Sternberg , E. Kaya , E. Ma , C. Anders , M. Hauer , K. Zhou , S. Lin , M. Kaplan , A. T. Iavarone , E. Charpentier , E. Nogales , J. A. Doudna Science. 2014, 343, 1247997. 24505130 10.1126/science.1247997 PMC4184034

[6] F. Jiang , J. A. Doudna , Annu. Rev. Biophys. 2017, 46, 505–529. 28375731 10.1146/annurev-biophys-062215-010822

[7] V. Pattanayak , S. Lin , J. P. Guilinger , E. Ma , J. A. Doudna , D. R. Liu , Nat. Biotechnol. 2013, 31, 839–843. 23934178 10.1038/nbt.2673 PMC3782611

[8] U. S. Kadam , R. M. Shelake , R. L. Chavhan , P. Suprasanna , Plant Physiol. Biochem. 2018, 131, 22–30. 29653762 10.1016/j.plaphy.2018.03.027

[9] M. Pacesa , C.-H. Lin , A. Cléry , A. Saha , P. R. Arantes , K. Bargsten , M. J. Irby , F. H. T. Allain , G. Palermo , P. Cameron , P. D. Donohoue , M. Jinek , Cell. 2022, 185, 4067–4081.e4021. 36306733 10.1016/j.cell.2022.09.026 PMC10103147

[10] C. A. Vakulskas , M. A. Behlke , Nucleic Acid Ther. 2019, 29, 167–174. 31107154 10.1089/nat.2019.0790 PMC6686686

[11] S. Bae , J. Park , J.-S. Kim , Bioinformatics 2014, 30, 1473–1475. 24463181 10.1093/bioinformatics/btu048 PMC4016707

[12] J.-P. Concordet , M. Haeussler , Nucleic Acids Res. 2018, 46, W242–W245. 29762716 10.1093/nar/gky354 PMC6030908

[13] F. Heigwer , G. Kerr , M. Boutros , Nat. Methods. 2014, 11, 122–123. 24481216 10.1038/nmeth.2812

[14] I. M. Slaymaker , L. Gao , B. Zetsche , D. A. Scott , W. X. Yan , F. Zhang , Science. 2016, 351, 84. 26628643 10.1126/science.aad5227 PMC4714946

[15] B. P. Kleinstiver , V. Pattanayak , M. S. Prew , S. Q. Tsai , N. T. Nguyen , Z. Zheng , J. K. Joung , Nature. 2016, 529, 490–495. 26735016 10.1038/nature16526 PMC4851738

[16] P. I. Kulcsár , A. Tálas , E. Tóth , A. Nyeste , Z. Ligeti , Z. Welker , E. Welker , Nat. Commun. 2020, 11, 1223. 32144253 10.1038/s41467-020-15021-5 PMC7060260

[17] J. K. Lee , E. Jeong , J. Lee , M. Jung , E. Shin , Y.-H. Kim , K. Lee , I. Jung , D. Kim , S. Kim , J.-S. Kim Nat. Commun. 2018, 9, 3048. 30082838 10.1038/s41467-018-05477-x PMC6078992

[18] H. K. Kim , S. Lee , Y. Kim , J. Park , S. Min , J. W. Choi , T. P. Huang , S. Yoon , D. R. Liu , H. H. Kim , Nat. Biomed. Eng. 2020, 4, 111–124. 31937939 10.1038/s41551-019-0505-1

[19] J. Shin , F. Jiang , J.-J. Liu , N. L. Bray , B. J. Rauch , S. H. Baik , E. Nogales , J. Bondy-Denomy , J. E. Corn , J. A. Doudna , Sci. Adv. 2017, 3, e1701620. 28706995 10.1126/sciadv.1701620 PMC5507636

[20] S. Aschenbrenner , S. M. Kallenberger , M. D. Hoffmann , A. Huck , R. Eils , D. Niopek , Sci. Adv. 2020, 6, eaay0187. 32076642 10.1126/sciadv.aay0187 PMC7002122

[21] Y. Fu , J. D. Sander , D. Reyon , V. M. Cascio , J. K. Joung , Nat. Biotechnol. 2014, 32, 279–284. 24463574 10.1038/nbt.2808 PMC3988262

[22] D. D. Kocak , E. A. Josephs , V. Bhandarkar , S. S. Adkar , J. B. Kwon , C. A. Gersbach , Nat. Biotechnol. 2019, 37, 657–666. 30988504 10.1038/s41587-019-0095-1 PMC6626619

[23] D. E. Ryan , D. Taussig , I. Steinfeld , S. M. Phadnis , B. D. Lunstad , M. Singh , X. Vuong , K. D. Okochi , R. McCaffrey , M. Olesiak , S. Roy , C. W. Yung , B. Curry , J. R. Sampson , L. Bruhn , D. J. Dellinger , Nucleic Acids Res. 2018, 46, 792–803. 29216382 10.1093/nar/gkx1199 PMC5778453

[24] H. Yin , C.-Q. Song , S. Suresh , S.-Y. Kwan , Q. Wu , S. Walsh , J. Ding , R. L. Bogorad , L. J. Zhu , S. A. Wolfe , V. Koteliansky , W. Xue , R. Langer , D. G. Anderson , Nat. Chem. Biol. 2018, 14, 311–316. 29377001 10.1038/nchembio.2559 PMC5902734

[25] P. D. Donohoue , M. Pacesa , E. Lau , B. Vidal , M. J. Irby , D. B. Nyer , T. Rotstein , L. Banh , M. S. Toh , J. Gibson , B. Kohrs , K. Baek , A. L. G. Owen , E. M. Slorach , M. van Overbeek , C. K. Fuller , A. P. May , M. Jinek , P. Cameron , Mol. Cell. 2021, 81, 3637–3649.e3635. 34478654 10.1016/j.molcel.2021.07.035

[26] X. Wu , D. A. Scott , A. J. Kriz , A. C. Chiu , P. D. Hsu , D. B. Dadon , A. W. Cheng , A. E. Trevino , S. Konermann , S. Chen , R. Jaenisch , F. Zhang , P. A. Sharp , Nat. Biotechnol. 2014, 32, 670–676. 24752079 10.1038/nbt.2889 PMC4145672

[27] J. N. Zadeh , C. D. Steenberg , J. S. Bois , B. R. Wolfe , M. B. Pierce , A. R. Khan , R. M. Dirks , N. A. Pierce , J. Comput. Chem. 2011, 32, 170–173. 20645303 10.1002/jcc.21596

[28] H. Nishimasu , F. A. Ran , P. D. Hsu , S. Konermann , S. I. Shehata , N. Dohmae , R. Ishitani , F. Zhang , O. Nureki , Cell. 2014, 156, 935–949. 24529477 10.1016/j.cell.2014.02.001 PMC4139937

[29] S. B. Thyme , L. Akhmetova , T. G. Montague , E. Valen , A. F. Schier , Nat. Commun. 2016, 7, 11750. 27282953 10.1038/ncomms11750 PMC4906408

[30] D. A. Specht , Y. Xu , G. Lambert , Proc. Nat. Acad. Sci. 2020, 117, 11274–11282. 32376630 10.1073/pnas.1918685117 PMC7260945

[31] B. Eslami-Mossallam , M. Klein , C. V. D. Smagt , K. V. D. Sanden , S. K. Jones , J. A. Hawkins , I. J. Finkelstein , M. Depken , Nat. Commun. 2022, 13, 1367. 35292641 10.1038/s41467-022-28994-2 PMC8924176

[32] E. A. Boyle , J. O. L. Andreasson , L. M. Chircus , S. H. Sternberg , M. J. Wu , C. K. Guegler , J. A. Doudna , W. J. Greenleaf , Proc. Nat. Acad. Sci. 2017, 114, 5461–5466. 28495970 10.1073/pnas.1700557114 PMC5448226

[33] E. A. Josephs , D. D. Kocak , C. J. Fitzgibbon , J. McMenemy , C. A. Gersbach , P. E. Marszalek , Nucleic Acids Res. 2015, 43, 8924–8941. 26384421 10.1093/nar/gkv892 PMC4605321

[34] L. Ratmeyer , R. Vinayak , Y. Y. Zhong , G. Zon , W. D. Wilson , Biochemistry. 1994, 33, 5298–5304. 7513557 10.1021/bi00183a037

[35] E. A. Lesnik , S. M. Freier , Biochemistry. 1995, 34, 10807–10815. 7662660 10.1021/bi00034a013

[36] J. I. Gyi , G. L. Conn , A. N. Lane , T. Brown , Biochemistry. 1996, 35, 12538–12548. 8823191 10.1021/bi960948z

[37] J. I. Gyi , A. N. Lane , G. L. Conn , T. Brown , Nucleic Acids Res. 1998, 26, 3104–3110. 9628906 10.1093/nar/26.13.3104 PMC147665

[38] J. Zhao , R. Inomata , Y. Kato , M. Miyagishi , Nucleic Acids Res. 2021, 49, 1330–1344. 33123724 10.1093/nar/gkaa865 PMC7897479

[39] H. Kim , W.-J. Lee , Y. Oh , S.-H. Kang , J. K. Hur , H. Lee , W. Song , K.-S. Lim , Y.-H. Park , B.-S. Song , Y. B. Jin , B.-H. Jun , C. Jung , D.-S. Lee , S.-U. Kim , S. H. Lee , Nucleic Acids Res. 2020, 48, 8601–8616. 32687187 10.1093/nar/gkaa605 PMC7470973

[40] S. Kim , D. Kim , S. W. Cho , J. Kim , J. S. Kim , Genome Res. 2014, 24, 1012–1019. 24696461 10.1101/gr.171322.113 PMC4032847

[41] E. A. Stadtmauer , J. A. Fraietta , M. M. Davis , A. D. Cohen , K. L. Weber , E. Lancaster , P. A. Mangan , I. Kulikovskaya , M. Gupta , F. Chen , L. Tian , V. E. Gonzalez , J. Xu , I.-Y. Jung , J. J. Melenhorst , G. Plesa , J. Shea , T. Matlawski , A. Cervini , A. L. Gaymon , S. Desjardins , A. Lamontagne , J. Salas-Mckee , A. Fesnak , D. L. Siegel , B. L. Levine , J. K. Jadlowsky , R. M. Young , A. Chew , W.-T. Hwang , E. O. Hexner , B. M. Carreno , C. L. Nobles , F. D. Bushman , K. R. Parker , Y. Qi , A. T. Satpathy , H. Y. Chang , Y. Zhao , S. F. Lacey , C. H. June , Science. 2020, 367, eaba7365.32029687

[42] L. Xu , J. Wang , Y. Liu , L. Xie , B. Su , D. Mou , L. Wang , T. Liu , X. Wang , B. Zhang , L. Zhao , L. Hu , H. Ning , Y. Zhang , K. Deng , L. Liu , X. Lu , T. Zhang , J. Xu , C. Li , H. Wu , H. Deng , H. Chen N Engl , J. Med. 2019, 381, 1240–1247. 10.1056/NEJMoa1817426 31509667

[43] Y. Rui , D. R. Wilson , J. Choi , M. Varanasi , K. Sanders , J. Karlsson , M. Lim , J. J. Green , Sci. Adv. 2019, 5, eaay3255. 31840076 10.1126/sciadv.aay3255 PMC6897553

[44] D. N. Nguyen , T. L. Roth , P. J. Li , P. A. Chen , R. Apathy , M. R. Mamedov , L. T. Vo , V. R. Tobin , D. Goodman , E. Shifrut , J. A. Bluestone , J. M. Puck , F. C. Szoka , A. Marson , Nat. Biotechnol. 2020, 38, 44–49. 31819258 10.1038/s41587-019-0325-6 PMC6954310

[45] V. Madigan , F. Zhang , J. E. Dahlman , Nat. Rev. Drug Discovery 2023, 22, 875–894. 37723222 10.1038/s41573-023-00762-x

[46] D. Wilbie , J. Walther , E. Mastrobattista , Acc. Chem. Res. 2019, 52, 1555–1564. 31099553 10.1021/acs.accounts.9b00106 PMC6584901

[47] C. A. Schneider , W. S. Rasband , K. W. Eliceiri , Nat. Methods. 2012, 9, 671–675. 22930834 10.1038/nmeth.2089 PMC5554542

