# Supplementary material for: Modulation of CRISPR‐Cas9 Cleavage with an Oligo‐Ribonucleoprotein Design
Source: Chembiochem. 2025 Jan 20;26(4):e202400821. doi: 10.1002/cbic.202400821 (PMC11833760; doi:10.1002/cbic.202400821)
Supplement: Supplementary file 1 — Supporting Information [file CBIC-26-e202400821-s001.pdf]

ChemBioChem

Supporting Information

Modulation of CRISPR-Cas9 Cleavage with an Oligo-Ribonucleoprotein Design

Yahui Gao, Yan Shan Ang, and Lin-Yue Lanry Yung*

Supporting Information

Modulation of CRISPR-Cas9 cleavage with an oligo-ribonucleoprotein design

Yahui Gao, Yan Shan Ang and Lin-Yue Lanry Yung*

Department of Chemical & Biomolecular Engineering, National University of Singapore, Singapore 117585, Singapore

*Corresponding Author

Email addresses: cheyly@nus.edu.sg

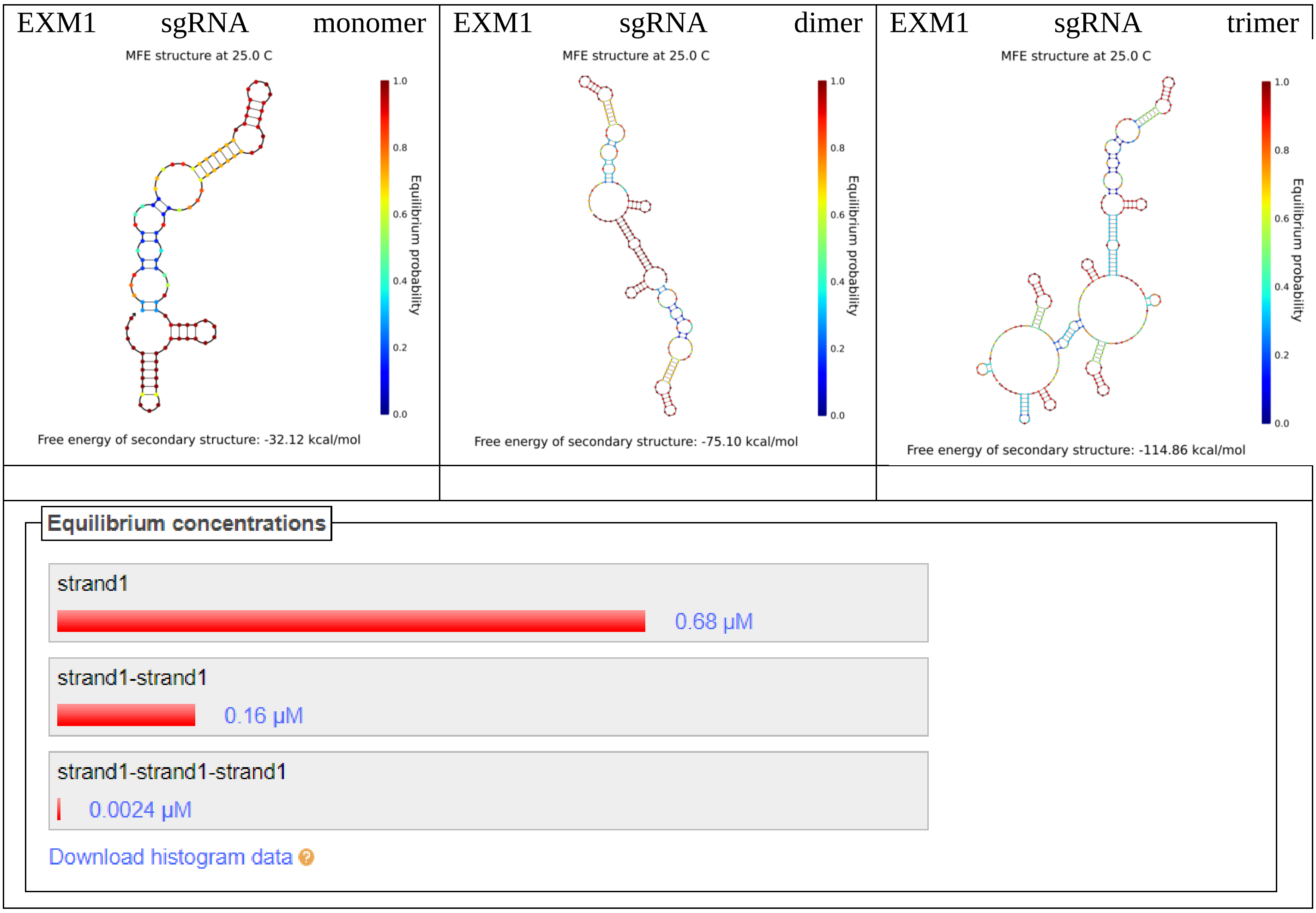

Fig. S1. NUPACK simulation of EXM1 sgRNA monomer, dimer, and trimer. The simulation condition was set at 1μM of EXM1 sgRNA at 25°C. The equilibrium concentrations of the 3 forms were shown. The ratio of equilibrium concentration of sgRNA monomer represented by strand 1 to that of sgRNA dimer (strand 1-strand 1) is about 4.25 :1. Very little concentration of sgRNA trimer (strand1-strand1-strand1) can also be formed.

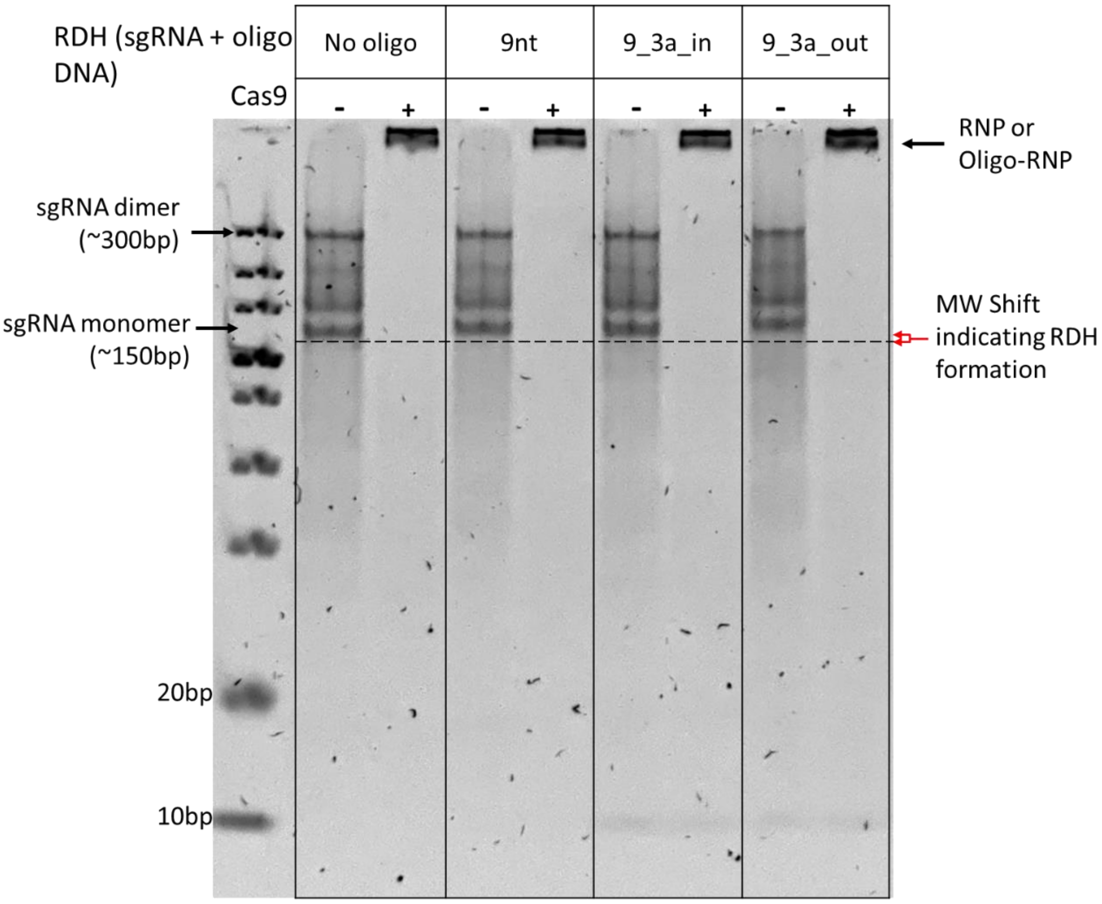

Fig. S2. Formation of oligo-RNP using the proposed assembly method. Gel electrophoresis image of sgRNA, sgRNA with RDH or oligo-RNP complex formed. 9nt, 9nt_3a_in, 9nt_3a_out represent different oligo designs used in formation of RDH with sgRNA. "No oligo" means no oligo was added. "–"and "+" represent conditions without Cas9 or with Cas9 added. In the conditions where Cas9 was not added ("–"), sgRNA dimer refers to the dimer structure formed by 2 sgRNA monomers via hybridization between their secondary structures (non-spacer regions). The shift in molecular weight (MW) of RDH formed via hybridization of oligo DNA and sgRNA is indicated by arrows in red. When Cas9 was added ("+"), the bands on top were indicated as RNP or oligo-RNP formed by RDH and Cas9.

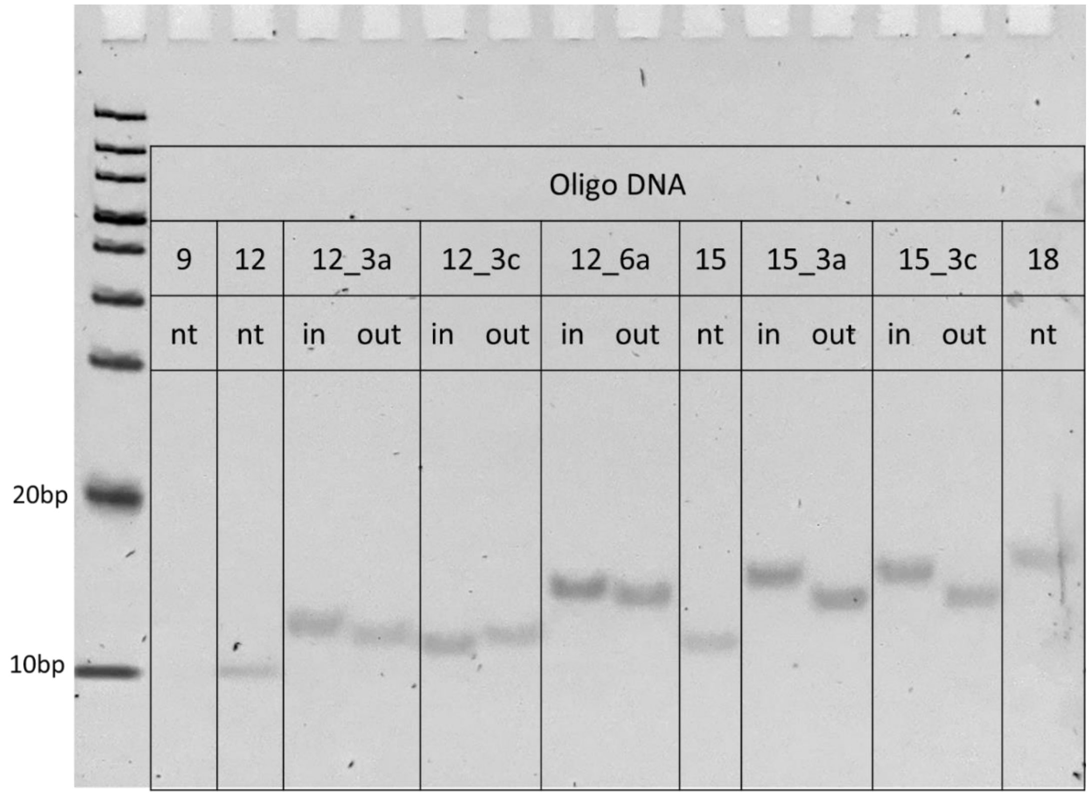

Fig. S3. SYBR gold staining for oligo single stranded DNA strands for EXM1 sgRNA. All oligos were prepared at the same concentration of 0.5µM. Note that the SYBR Gold staining intensity is highly dependent on the number of nucleotides therefore the band intensity of oligo DNA 9nt was too low to be seen on the gel and the band intensity was observed to be higher for those oligo DNA with longer total length of nucleotides. Previous study has also shown that SYBR Gold stains single-stranded oligo DNA much less efficiently than double-stranded DNA.[1, 2]

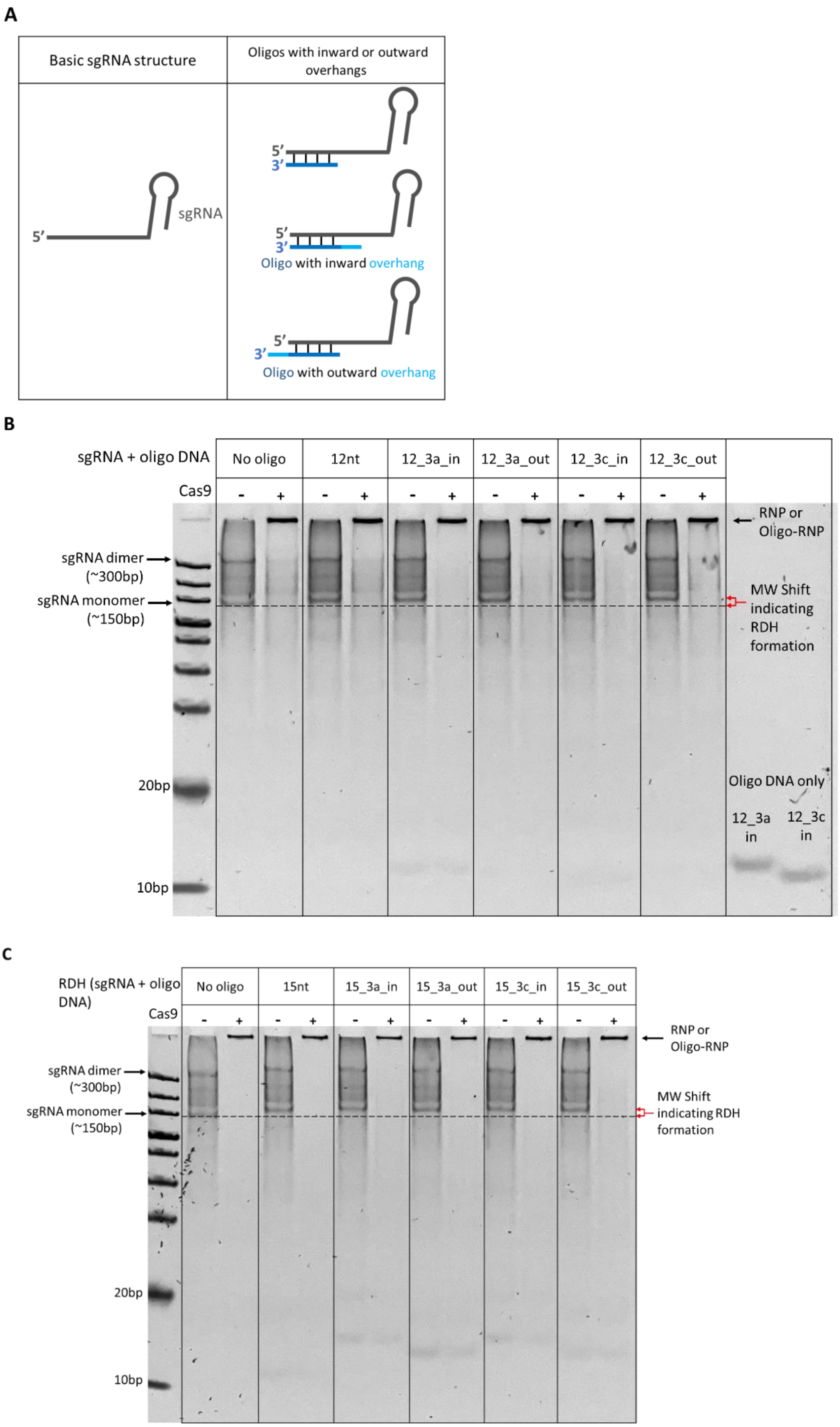
A
Basic sgRNA structure
Oligos with inward or outward overhangs
5'
sgRNA
5'
3'
5'
3'
Oligo with inward overhang
5'
3'
Oligo with outward overhang
B
sgRNA + oligo DNA
No oligo
12nt
12_3a_in
12_3a_out
12_3c_in
12_3c_out
Cas9
- + - + - + - + - + - +
RNP or Oligo-RNP
sgRNA dimer (~300bp)
sgRNA monomer (~150bp)
MW Shift indicating RDH formation
20bp
10bp
Oligo DNA only
12_3a in
12_3c in
C
RDH (sgRNA + oligo DNA)
No oligo
15nt
15_3a_in
15_3a_out
15_3c_in
15_3c_out
Cas9
- + - + - + - + - + - +
RNP or Oligo-RNP
sgRNA dimer (~300bp)
sgRNA monomer (~150bp)
MW Shift indicating RDH formation
20bp
10bp

Fig. S4. (A) Schematic illustration of basic sgRNA (dark grey) and proposed oligo-sgRNA designs with adding inward or outward overhangs (light blue) onto the oligo DNA (dark blue). Inward overhang refers to non-complementary overhang sequence added at the 5' end of oligo DNA and proximal to non-spacer region of the sgRNA. Outward overhang means the non-complementary overhang is located at the 3' end of oligo DNA and distal to the non-spacer region of sgRNA (near 5' end of sgRNA). (**B)** Gel electrophoresis image of sgRNA, sgRNA with RDH or oligo-RNP complex formed. 12nt, 12nt_3a_in, 12nt_3a_out, 12nt_3c_in and 12nt_3c_out represent different oligo designs used in formation of RDH with sgRNA. (**C)** Gel electrophoresis image of sgRNA, sgRNA with RDH or oligo-RNP complex formed. 15nt, 15nt_3a_in, 15nt_3a_out, 15nt_3c_in and 15nt_3c_out represent different oligo designs used in formation of RDH with sgRNA. "No oligo" means no oligo was added. "–"and "+" represent conditions without Cas9 or with Cas9 added. In the conditions where Cas9 was not added ("–"), sgRNA dimer refers to the dimer structure formed by 2 sgRNA monomers via hybridization between their secondary structures (non-spacer regions). The shift in molecular weight (MW) of RDH formed via hybridization of oligo DNA and sgRNA is indicated by arrows in red. When Cas9 was added ("+"), the bands on top were indicated as RNP or oligo-RNP formed by RDH and Cas9. Note that in Supplementary Fig. S4C there were faint oligo DNA bands present on the gel in the range between 10bp and 20bp in both conditions with and without Cas9. This is attributed to an excess amount of oligo DNA present compared to sgRNA since the band intensity was much lower than those of oligo DNA alone (Fig. S3) and the band intensity did not increase after the addition of Cas9.

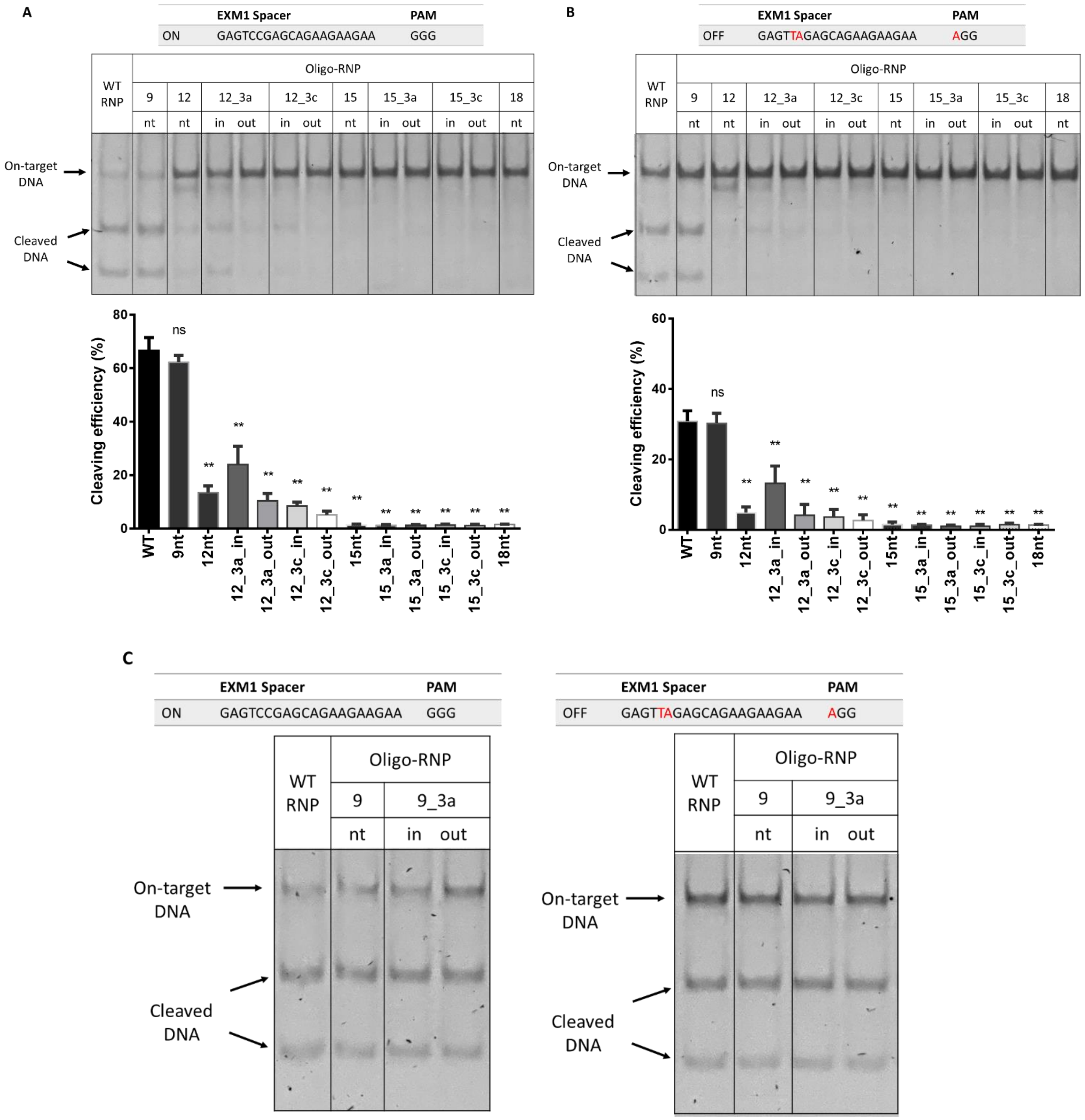

Fig. S5. In vitro EXM1 on-target and off-target cleaving assay results using oligo DNA designs with and without overhangs. **(A)** Representative gel electrophoresis image and the corresponding quantification of the in vitro on-target cleaving efficiencies of oligo-RNPs. The sequence of the on-target DNA was shown in the table on top. **(B)** Representative gel electrophoresis image and the corresponding quantification of the in vitro off-target cleaving efficiency. The sequence of the off-target DNA was shown in the table on top. Cleavage efficiency was assessed by gel electrophoresis and measured using densitometry (%).[3] All

data shown are mean ± standard deviation (SD). Dots are individual data points from independent experiments (n = 3). Statistical analysis was done using two-tailed unpaired Student's t-test of comparison between WT and oligo-RNP of each oligo design. **denotes p < 0.01. WT RNP is unmodified RNP without oligo DNA. (**C**) Oligo-RNP with oligo DNA 9nt and its overhang variants (9nt_3a_in, 9nt_3a_out, 9nt_6a_in, 9nt_6a_out) did not reduce on-target and off-target cleaving efficiency (ns).

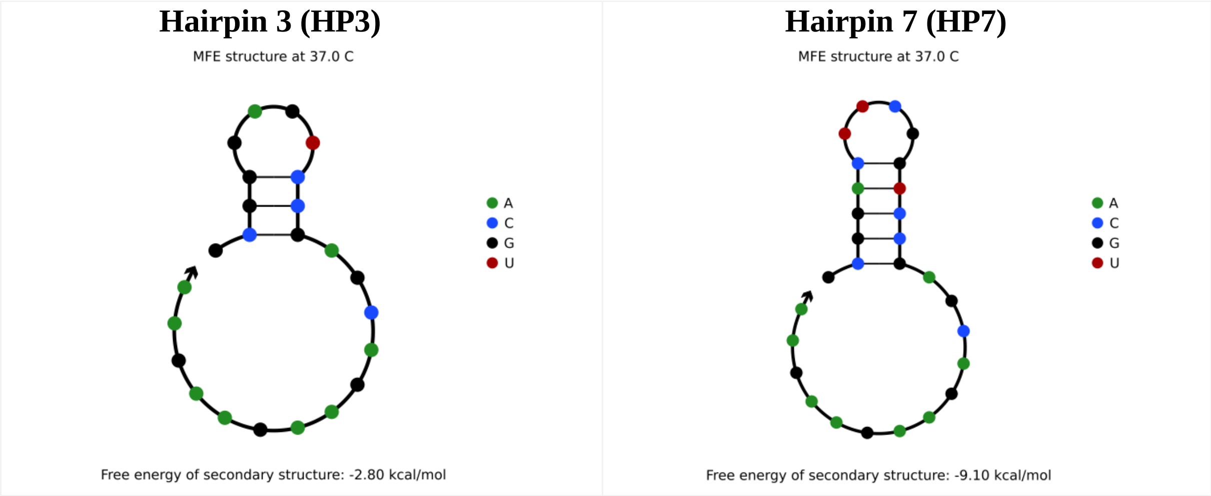

Fig. S6. Spacer structures of Hairpin 3 and 7 (HP3 & HP7) sgRNA simulated by NUPACK for EXM1.

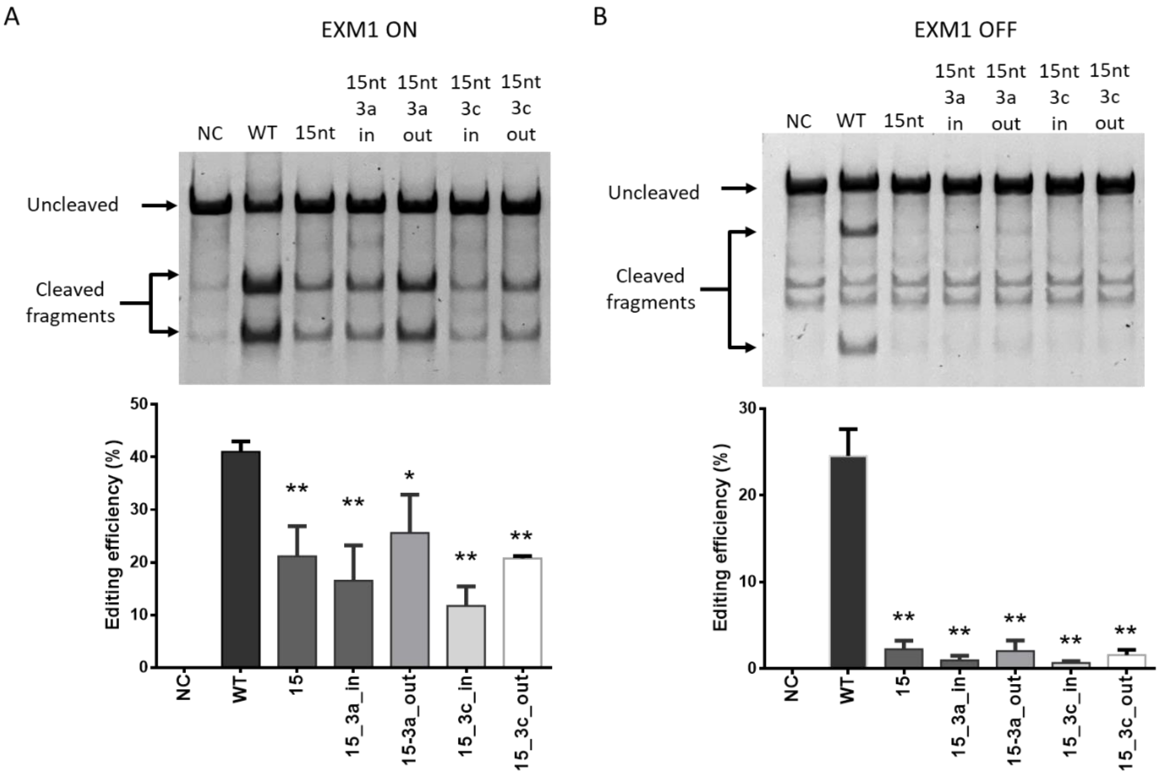

Fig. S7. On-target and off-target editing efficiencies for EXM1 using oligo-RNPs with the same oligo 15nt complementary sequence to sgRNA spacer with or without overhangs in HEK293 cells. (**A)** Representative gel electrophoresis image and the corresponding quantification of the on-target (ON) editing efficiencies for EXM1 using WT RNP and oligo-RNPs with different oligo designs with or without overhangs. (**B)** Representative gel electrophoresis image and the corresponding quantification of the off-target (OFF) editing efficiencies for EXM1 using WT RNP and oligo-RNPs with different oligo designs with or without overhangs. All data shown are mean ± standard deviation (SD) (n = 3). * denotes $p<0.05$, ** denotes $p<0.01$ for one-way ANOVA comparison between WT and oligo-RNP of each oligo design (15nt, 15nt_3a_in, 15nt_3a_out, 15nt_3c_in or 15nt_3c_out). NC is negative control.

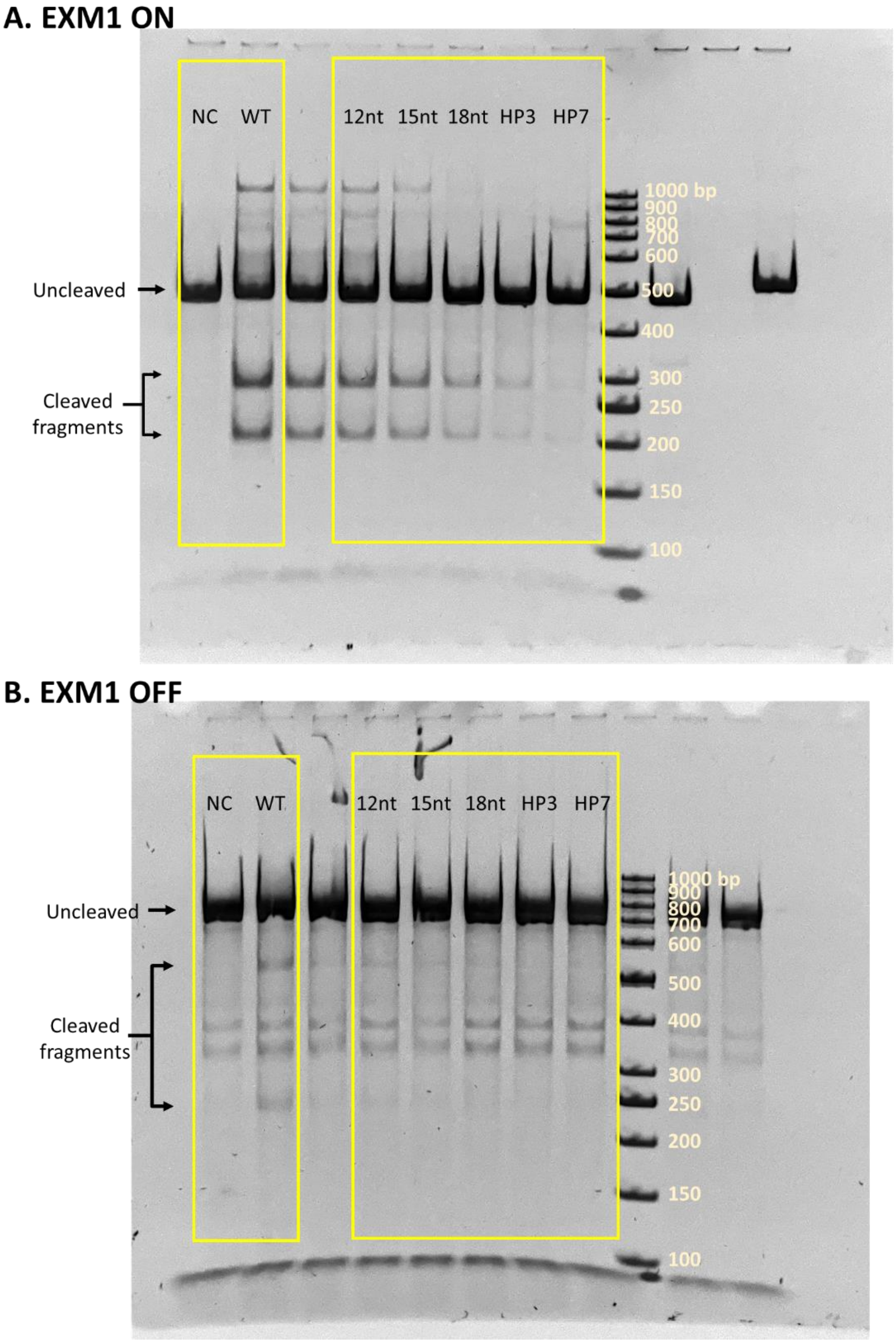

Fig. S8. Uncropped image to indicate the rearrangement of lanes in Fig. 4A and 4B. (A) Full gel image of Fig. 4A. (B) Full gel image of Fig. 4B.

A. VEGFA ON

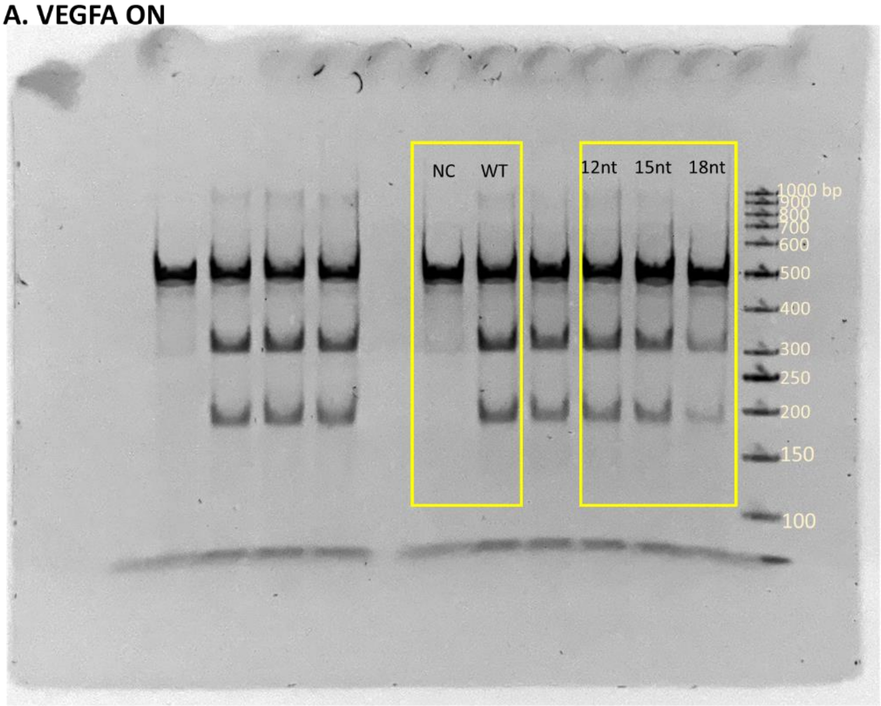

B. VEGFA OFF

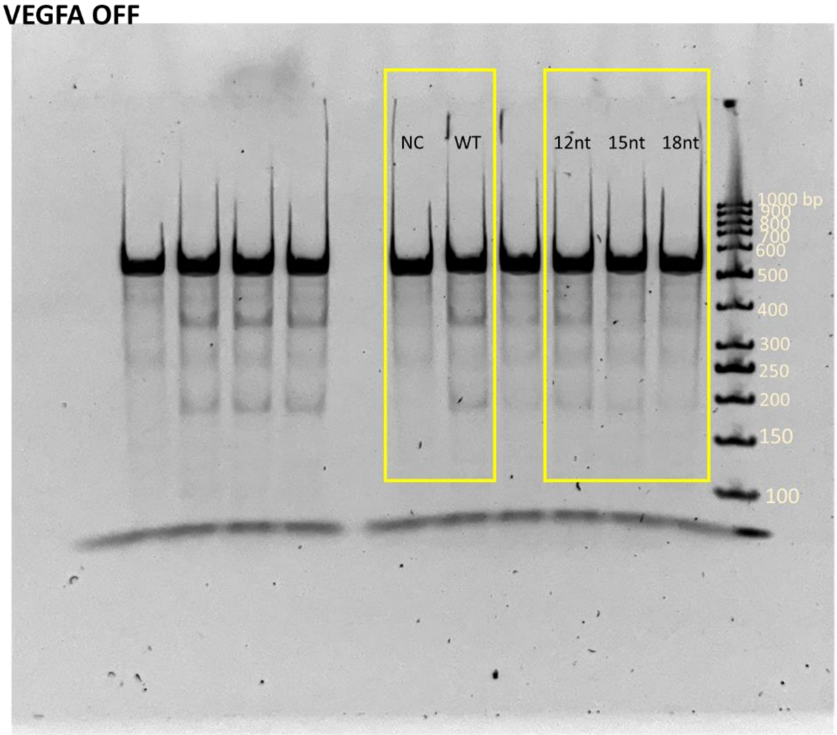

Fig. S9. Uncropped image to indicate the rearrangement of lanes in Fig. 5A and 5B. (A) Full gel image of Fig. 5A. (B) Full gel image of Fig. 5B.

A. HBB ON

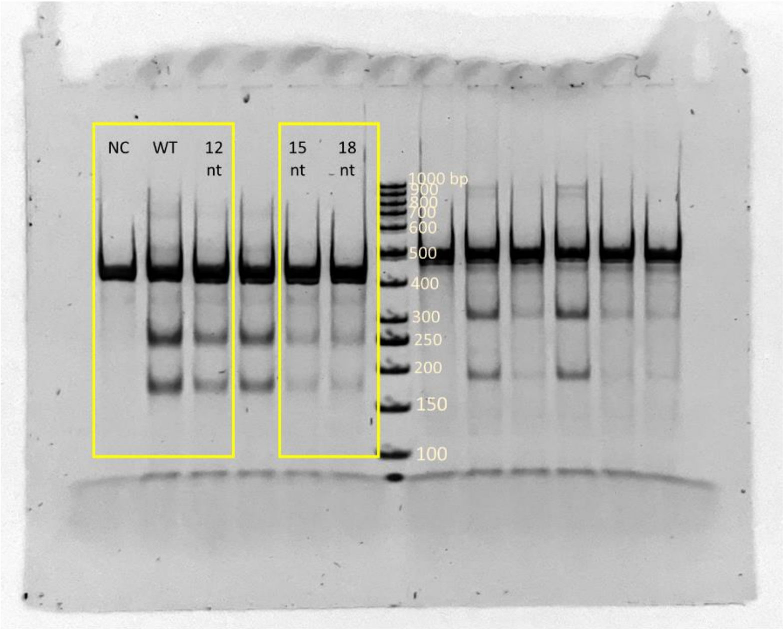

B. HBB ON

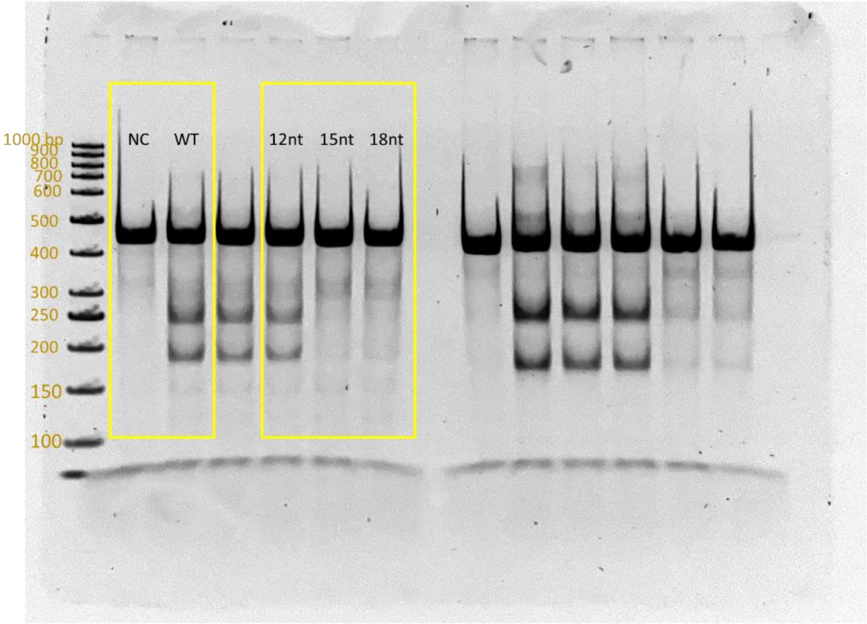

Fig. S10. Uncropped image to indicate the rearrangement of lanes in Fig. 5C and 5D. (A) Full gel image of Fig. 5C. (B) Full gel image of Fig. 5D.

Table S1. Sequences of DNA and sgRNA used in this study.

Oligo DNA		5' to 3'
EXM1	9nt	CTCGGACTC
	9nt_3a_in	AAA CTCGGACTC
	9nt_3a_out	CTCGGACTC AAA
	12nt	CTGCTCGGACTC
	12nt_3a_in	AAA CTGCTCGGACTC
	12nt_3a_out	CTGCTCGGACTC AAA
	12nt_3c_in	CCC CTGCTCGGACTC
	12nt_3c_out	CTGCTCGGACTC CCC
	15nt	CTTCTGCTCGGACTC
	15nt_3a_in	AAA CTTCTGCTCGGACTC
	15nt_3a_out	CTTCTGCTCGGACTC AAA
	15nt_3c_in	CCC CTTCTGCTCGGACTC
	15nt_3c_out	CTTCTGCTCGGACTC CCC
	18nt	CTTCTTCTGCTCGGACTC
VEGFA	12nt	CACTCACTCACC
	15nt	ACACACTCACTCACC
	18nt	CGCACACACTCACTCACC
HBB	12nt	CTGTGGGGCAAG
	15nt	GCCCTGTGGGGCAAG
	18nt	ACTGCCCTGTGGGGCAAG
EXM1	sgRNA	GAGUCCGAGCAGAAGAAGAA GUUUUAGAGCUAGAAAUAGCAAGUUAAAA UAAGGCUAGUCCGUUAUCAACUUGAAAAAGUGGCACCGAG UCGGUGCUUUU
EXM1 HP3	sgRNA	GCGG GAGUCCGAGCAGAAGAAGAA GUUUUAGAGCUAGAAAUAGCAAGUUAAAA UAAGGCUAGUCCGUUAUCAACUUGAAAAAGUGGCACCGAG UCGGUGCUUUU
EXM1 HP7	sgRNA	GCGGAC UUCG GUCCGAGCAGAAGAAGAA GUUUUAGAGCUAGAAAUAGCAAGUUAAAA UAAGGCUAGUCCGUUAUCAACUUGAAAAAGUGGCACCGA GUCGGUGCUUUU
VEGFA	sgRNA	GGUGAGUGAGUGUGUGCGUG GUUUUAGAGCUAGAAAUAGCAAGUUAAAA UAAGGCUAGUCCGUUAUCAACUUGAAAAAGUGGCACCGA GUCGGUGCUUUU
HBB	sgRNA	CUUGCCCCACAGGGCAGUAA GUUUUAGAGCUAGAAAUAGCAAGUUAAAA UAAGGCUAGUCCGUUAUCAACUUGAAAAAGUGGCACCG AGUCGGUGCUUUU
Synthetic target DNA for in vitro cleaving assay		
EXM1 ON	Target strand	GGAGGAGGAAGGGCCTGAGTCCGAGCA GAAGAAGAAGGGCTCCCATCACATCAAC

	Non-target strand	GTTGATGTGATGGGAGCCCTTCTTCTTCT GCTCGGACTCAGGCCCTTCCTCCTCC
EXM1 OFF	Target strand	GGAGGAGGAAGGGCCTGAGTTAGAGCA GAAGAAGAAAGGCTCCCATCACATCAAC
	Non-target strand	GTTGATGTGATGGGAGCCTTTCTTCTTCT GCTCTAACTCAGGCCCTTCCTCCTCC

Primers used for T7E1	5' to 3'	Amplicon size
EMX1 ON F<br><br>EMX1 ON R	TGGGGCCCCTAACCCTATGTA<br><br>AGAAGGCCAAGTGGTCCCAG	503bp
EMX1 OFF F<br><br>EMX1 OFF R	ACATGTCTTCCTCCAACTCTAACT<br><br>TCCTGAAGACCTGTAATCTGACTCT	634bp
VEGFA ON F<br><br>VEGFA ON R	TCCAGATGGCACATTGTCAG<br><br>AGGGAGCAGGAAAGTGAGGT	491bp
VEGFA OFF F<br><br>VEGFA OFF R	GCAGTTTTGGGTGTCTGTAGGG<br><br>TGGTAGTTGCCTGGGGATGG	500bp
HBB ON F<br><br>HBB ON R	CATCAGGAGTGGACAGATCCC<br><br>TAGGGTTGGCCAATCTACTCCC	425bp
HBB OFF F<br><br>HBB OFF R	GGAAATGCACTTTTCCAGATTCAGT<br><br>ACCTCCAGTTTCTAAGAGCGGT	403bp

Table S2. A summary of GC content and free energies of RDH formed by each oligo DNA used for EXM1, VEGFA and HBB and its corresponding sgRNA. The complementary parts to sgRNA spacer of the 4 main oligo lengths (9nt, 12nt, 15nt and 18nt) were compared. Free energy values for RNA-RNA duplex and DNA-DNA duplex were obtained from NUPACK with the assumption that the free energy of RDH would be between those of RNA-RNA and DNA-DNA. Oligo 15nt for the 3 targets are in bold for the ease of comparison.

	Length of oligo DNA sequence complementary to sgRNA spacer	GC content	Free energy (kcal/mol) at 37°C	
			RNA-RNA	DNA-DNA
EXM1	9nt	66.7%	-16.08	-12.65
	12nt	66.7%	-23.68	-18.31
	**15nt**	**66.7%**	**-28.58**	**-21.89**
	18nt	60.0%	-33.48	-25.47
VEGFA	9nt	55.6%	-16.18	-12.21
	12nt	58.3%	-21.58	-16.53
	**15nt**	**53.3%**	**-27.68**	**-20.96**
	18nt	61.1%	-34.78	-26.72
HBB	9nt	66.7%	-17.18	-13.43
	12nt	66.7%	-23.58	-18.06
	**15nt**	**73.3%**	**-33.18**	**-24.36**
	18nt	66.7%	-37.78	-28.14

References

[1] D. Y. Zhang, A. J. Turberfield, B. Yurke, E. Winfree *Science*. **2007**, *318*, 1121-1125.
[2] X. Han, E. Wang, Y. Cui, Y. Lin, H. Chen, R. An, X. Liang, M. Komiyama *Electrophoresis*. **2019**, *40*, 1708-1714.
[3] L. Cong, F. A. Ran, D. Cox, S. Lin, R. Barretto, N. Habib, P. D. Hsu, X. Wu, W. Jiang, L. A. Marraffini, F. Zhang *Science*. **2013**, *339*, 819-823.
